# The IDH1-R132H mutation aggravates cisplatin-induced acute kidney injury by promoting ferroptosis through disrupting NDUFA1 and FSP1 interaction

**DOI:** 10.1038/s41418-024-01381-8

**Published:** 2024-09-22

**Authors:** Kunmei Lai, Zhimin Chen, Siyi Lin, Keng Ye, Ying Yuan, Guoping Li, Yankun Song, Huabin Ma, Tak W. Mak, Yanfang Xu

**Affiliations:** 1 Department of Nephrology, Blood Purification Research Center, the First Affiliated Hospital, Fujian Medical University, Fuzhou, China; 2 Research Center for Metabolic Chronic Kidney Disease, the First Affiliated Hospital, Fujian Medical University, Fuzhou, China; 3 Department of Nephrology, National Regional Medical Center, Binhai Campus of the First Affiliated Hospital, Fujian Medical University, Fuzhou, China; 4 Department of Pathology, the First Affiliated Hospital, Fujian Medical University, Fuzhou, China; 5 Central Laboratory, the First Affiliated Hospital, Fujian Medical University, Fuzhou, China; 6 Princess Margaret Cancer Centre, Ontario Cancer Institute, University Health Network, Toronto, ON Canada; 7 Centre for Oncology and Immunology, Hong Kong Science Park, Hong Kong SA, China

**Keywords:** Kidney diseases, Disease genetics

## Abstract

The IDH1-R132H mutation is implicated in the development of various tumors. Whether cisplatin, a common chemotherapeutic agent, induces more significant renal toxicity in individuals with the IDH1-R132H mutation remains unclear. In this study, we observed that the IDH1-R132H mutation exacerbates mitochondrial lipid peroxidation and dysfunction in renal tubules, rendering the kidneys more susceptible to cisplatin-induced ferroptosis. The IDH1-R132H mutation increases methylation of the *Ndufa1* promoter, thereby suppressing NDUFA1 transcription and translation. This suppression disrupts NDUFA1’s interaction with FSP1, reducing its resistance to cisplatin-induced tubular epithelial cell death. As a consequence, ROS accumulates, lipid peroxidation occurs, and ferroptosis is triggered, thereby promoting acute kidney injury. In summary, this study elucidates a novel mechanism underlying cisplatin-induced nephrotoxicity and provides valuable insights for the development of personalized treatment strategies for tumor patients carrying the IDH1-R132H mutation.

## Introduction

Cisplatin, as a platinum-based chemotherapy drug, is one of the most widely used agents in current combined chemotherapy regimens. It demonstrates therapeutic efficacy in the treatment of various malignancies, including epithelial tumors, sarcomas, lymphomas, and germ cell tumors [1]. However, its administration is frequently accompanied by a range of side effects, such as nephrotoxicity, gastrointestinal reactions, neurological manifestations, hematological reactions [2]. These adverse effects limit the escalation of cisplatin dosage and its therapeutic effectiveness. Consequently, optimizing the efficacy of cisplatin, minimizing its adverse effects, and exploring novel applications and combination therapies to enhance its anticancer effects have emerged as significant challenges in clinical practice [3].

Acute kidney injury (AKI) is one of the most common adverse reactions associated with cisplatin, with approximately 30% of patients discontinuing therapy due to its nephrotoxicity [4]. The renal toxicity of cisplatin is dose-dependent; upon intravenous administration, it accumulates significantly within the kidneys, causing structural and functional damage, particularly to the renal tubules, leading to decreased renal function [5]. Studies have indicated that a cisplatin dosage of 10 mg/kg can induce mitochondrial dysfunction in the renal tubules of mice, resulting in deterioration of renal function and tubular necrosis [5, 6]. Currently, hydration therapy and diuresis are commonly recommended before and during high-dose cisplatin treatment to mitigate renal toxicity. However, there still lacks direct and effective therapeutic interventions to alleviate cisplatin-induced AKI (Cis-AKI) [7]. Therefore, exploring the pathogenic mechanisms underlying the reduction of cisplatin nephrotoxicity while harnessing its advantages in tumor therapy holds significant clinical significance.

One of the isoforms of isocitrate dehydrogenase (IDH), IDH1, plays a crucial role in maintaining the redox balance within the cellular oxidative state [8]. Under normal physiological conditions, wild-type IDH1 catalyzes the conversion of isocitrate to α-ketoglutarate (α-KG) and CO_2_ through reduction and oxidative decarboxylation reactions, concurrently reducing NADP+ to form NADPH as a cofactor [9–11]. Upon mutation, the catalytic activity of IDH1 is altered. The most common mutation in IDH1 occurs at residue 132, where arginine is replaced by histidine (IDH1-R132H) [12, 13]. This mutation leads to a loss of function in the wild-type IDH1 enzyme and the acquisition of a new activity, converting α-KG into the oncometabolite D-2-hydroxyglutarate (D-2HG), thereby consuming NADPH and enhancing cell death [12].

The IDH1-R132H mutation has been implicated in the occurrence and progression of tumors, potentially promoting tumorigenesis through alterations in cellular metabolic pathways and epigenetic regulation [14]. Prior studies have reported that the deletion of the IDH2 gene, one of the isoforms of isocitrate dehydrogenase, in ischemia-reperfusion injury (IRI) and cisplatin-induced acute kidney injury results in decreased NADPH levels, reduced GPX activity, and mitochondrial dysfunction in renal tubular cells [15–17]. This leads to increased oxidative stress and severe apoptosis in these cells, ultimately exacerbating renal function deterioration. Given the correlation between the IDH1-R132H mutation and tumorigenesis, and considering cisplatin’s status as a commonly used chemotherapy drug, it remains unclear whether cisplatin would induce more pronounced renal toxicity in individuals with the IDH1-R132H mutation [18, 19].

Therefore, this study aims to gain deeper insights into the mechanism of cisplatin-induced nephrotoxicity and investigate, through a series of in vivo and in vitro experiments, whether the IDH1-R132H mutation exacerbates cisplatin-induced tubular epithelial cell injury by affecting mitochondrial oxidative stress, thereby promoting the occurrence of cis-AKI. This research aims to provide more personalized treatment strategies for tumor patients carrying the IDH1-R132H mutation to reduce the occurrence of kidney injury.

## Results

### The IDH1-R132H mutation in renal tubules exacerbated cisplatin-induced renal dysfunction

**Fig. 1:**
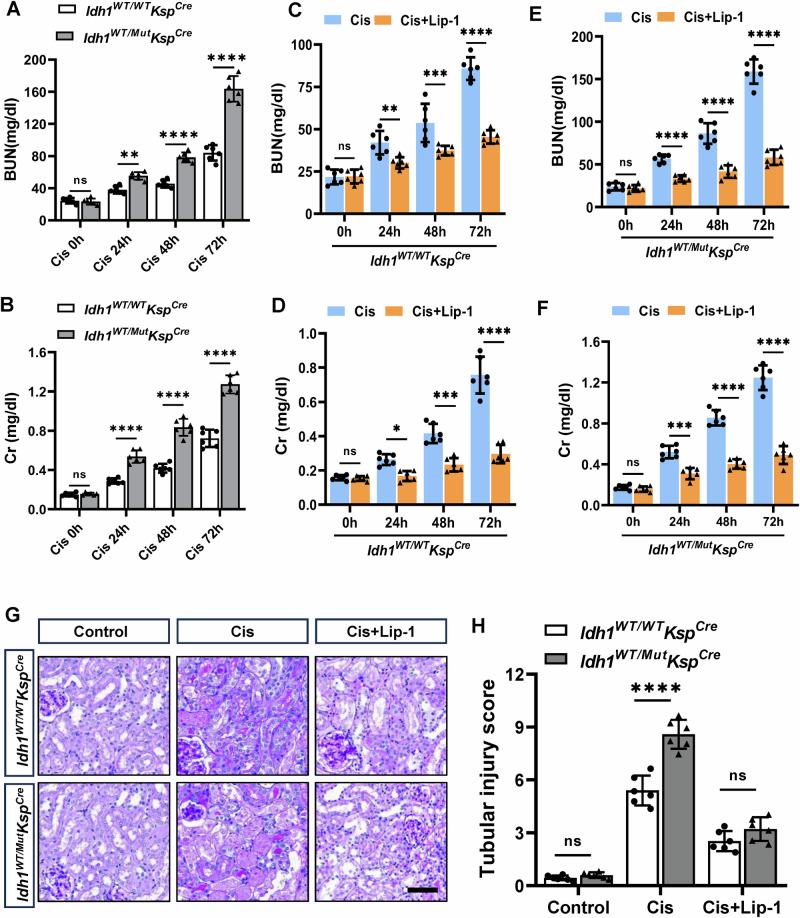
IDH1-R132H mutation exacerbated cisplatin-induced renal dysfunction in mice. Measurement of blood urea nitrogen (BUN) (**A**) and serum creatinine (Scr) levels (**B**) at 0 h, 24 h, 48 h, and 72 h after cisplatin administration. The Scr and BUN levels in *Idh1*^*WT/Mut*^*Ksp*^*Cre*^ mice were higher than those in the *Idh1*^*WT/WT*^*Ksp*^*Cre*^ group. **C**–**F** Pretreatment with the ferroptosis inhibitor Lip-1 at 1 day and 2 h before intraperitoneal injection of 10 mg/kg cisplatin was administered. Changes in BUN and creatinine levels were observed at 0 h, 24 h, 48 h, and 72 h. ns not significant; *P < 0.05; **P < 0.01; ***P < 0.001; ****P < 0.0001. **G**, **H** Representative images of PAS-stained kidney sections after intraperitoneal injection of 10 mg/kg cisplatin for 3 days, Scale bar = 100 μM. **H** Quantitative analysis of renal injury outcomes in (**G**).

To investigate the role of the IDH1-R132H mutation in renal injury, we used specific mutation of IDH1 in renal tubules (*Idh1*^*WT/Mut*^*Ksp*^*Cre*^) in this study. *Idh1*^*WT/Mut*^*Ksp*^*Cre*^ mice exhibited significantly elevated levels of blood urea nitrogen (BUN) and serum creatinine (Scr) compared to *Idh1*^*WT/WT*^*Ksp*^*Cre*^ mice, indicating progressive renal injury (Fig. 1A, B). Notably, compared to the apoptosis inhibitor zVAD, the ferroptosis inhibitor Lip-1, significantly reduced BUN and Scr levels in both *Idh1*^*WT/WT*^*Ksp*^*Cre*^ and *Idh1*^*WT/Mut*^*Ksp*^*Cre*^ mice (Fig. 1C–F, S1). Histological analysis revealed that *Idh1*^*WT/Mut*^*Ksp*^*Cre*^ mice sustained more severe renal tissue damage following cisplatin treatment compared to *Idh1*^*WT/WT*^*Ksp*^*Cre*^ mice, characterized by increased tubular epithelial cell necrosis, swelling, and numerous protein casts. In contrast, Lip-1 pretreatment ameliorated renal tissue damage (Fig. 1G, H). These findings suggested that the IDH1-R132H mutation in renal tubules exacerbated cisplatin-induced renal dysfunction, potentially through activation of ferroptosis-related signaling pathways.

### IDH1-R132H mutation aggravated cisplatin-induced oxidative stress and proximal tubular epithelial cell ferroptosis

**Fig. 2:**
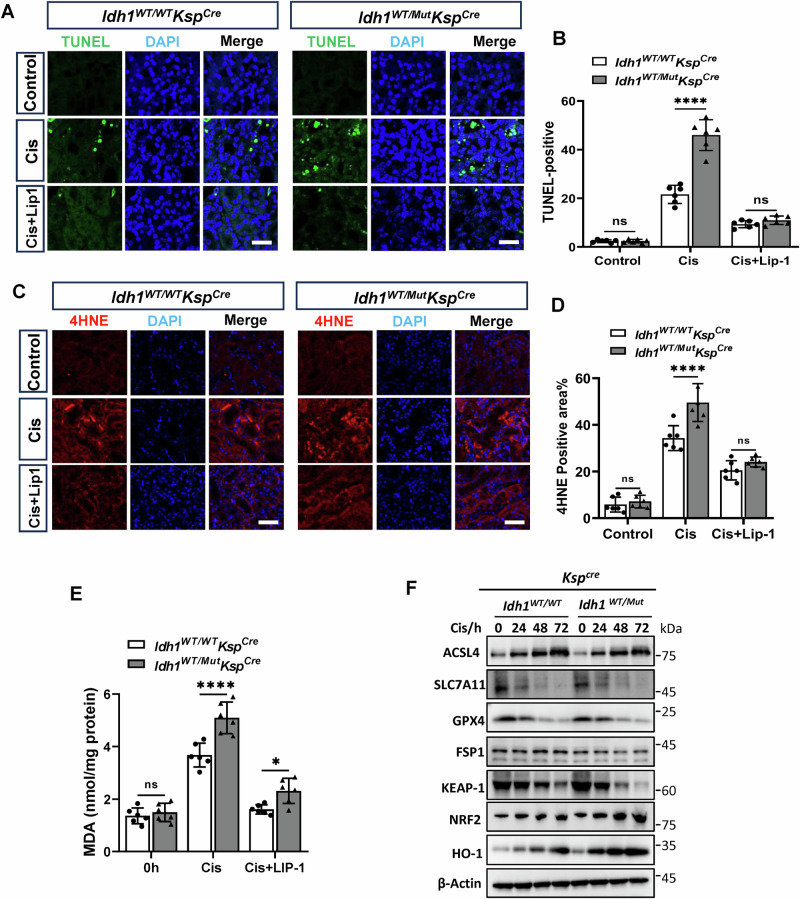
IDH1-R132H mutation aggravated cisplatin-induced oxidative stress and tubular epithelial cell ferroptosis. **A**, **B** Quantitative analysis and representative staining images of TUNEL in the kidney. The TUNEL-positive PTCs in the *Idh1*^*WT/Mut*^*Ksp*^*Cre*^ group was higher than that in the *Idh1*^*WT/WT*^*Ksp*^*Cre*^ group, *n* = 6, ns not significant, ****P < 0.0001. **C**, **D** Immunofluorescence staining of 4HNE (red) and DAPI (blue) in kidneys injected intraperitoneally with 10 mg/kg cisplatin for 3 days, followed by quantification of 4HNE-positive cells. *n* = 6, ns *n*ot significant, ****P < 0.0001. **E** Quantification of MDA content of the kidney at 3 days after intraperitoneal injection with 10 mg/kg cisplatin. *n* = 6. ns not significant, *P < 0.05, **P < 0.01, ***P < 0.001. **F** Fresh proximal tubules from the kidneys were harvested for western blot analysis, focusing on the expression of ferroptotic and antioxidant signaling molecules. β-actin was used as a loading control. *n* = 4.

Previous studies have confirmed that proximal tubular epithelial cells **(**PTCs) are the primary targets of Cis-AKI. We explored the specific mechanisms of PTC injury induced by the IDH1-R132H mutation. TUNEL staining revealed that the IDH1-R132H mutation significantly exacerbates cisplatin-induced PTC death, while Lip-1 pretreatment alleviates this cell death (Fig. 2A, B). Furthermore, 4-HNE and MDA, the lipid peroxidation products were evaluated in kidney tissues of *Idh1*^*WT/WT*^*Ksp*^*Cre*^ and *Idh1*^*WT/Mut*^*Ksp*^*Cre*^ mice 72 h post-cisplatin treatment. The oxidative stress injury levels were significantly higher in the kidneys of the *Idh1*^*WT/Mut*^*Ksp*^*Cre*^ group compared to the *Idh1*^*WT/WT*^*Ksp*^*Cre*^ group. Lip-1 pretreatment significantly mitigated renal oxidative stress injury (Fig. 2C–E). We further confirmed oxidative stress and ferroptosis-related protein expression in the proximal tubules isolated from kidney tissues. The IDH1-R132H mutation led to increased expression of oxidative stress-related proteins NRF2 and HO-1 and decreased KEAP-1 expression in cisplatin-induced tubular injury. Compared to *Idh1*^*WT/WT*^*Ksp*^*Cre*^ mice, cisplatin-treated *Idh1*^*WT/Mut*^*Ksp*^*Cre*^ mice exhibited increased expression of the core ferroptosis protein ACSL4 and decreased expression of GPX4 and SLC7A11 (Fig. 2F). These findings provide compelling evidence that the IDH1-R132H mutation exacerbates Cis-AKI by promoting ferroptosis.

### The IDH1-R132H mutation induced ferroptosis by promoting mitochondrial dysfunction

**Fig. 3:**
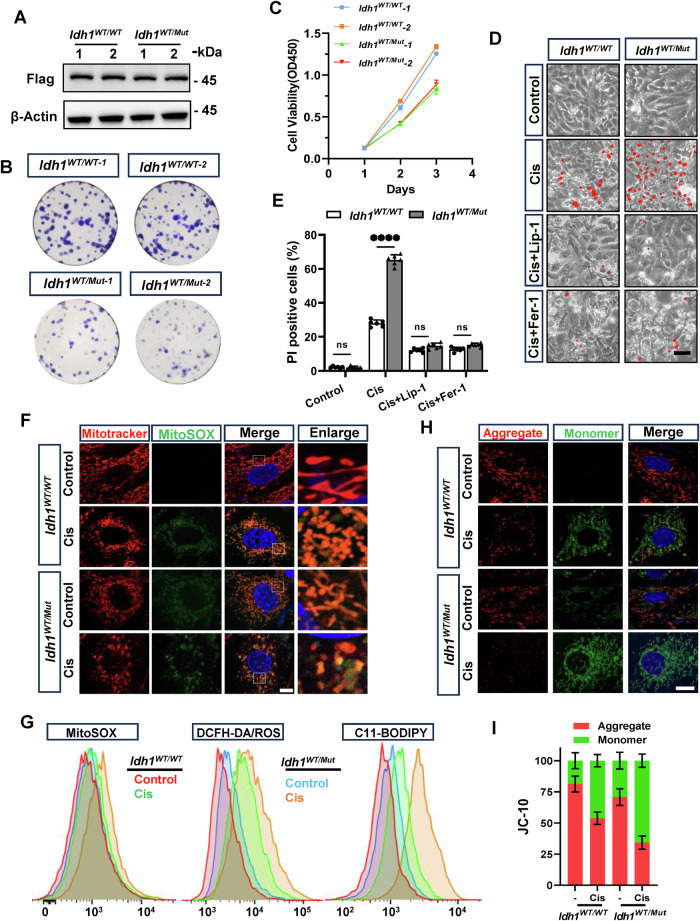
The IDH1-R132H mutation induces ferroptosis by promoting mitochondrial dysfunction. **A** TECs (cell line) were infected with lentivirus encoding Flag-IDH1-WT and Flag-IDH1-R132H for 36 h, followed by stimulation with 25 μm cisplatin for 24 h. Western blot was performed to detect IDH1 expression after 36 h of infection. **B**, **C** TECs from (**A**) were seeded in 12-well plates. Colony counting was conducted on the 10th day after seeding. Cell proliferation was measured using the CCK-8 assay (**C**). **D**–**I** PTCs were isolated from *Idh1*^*WT/Wt*^*Ksp*^*Cre*^ and *Idh1*^*WT/Mut*^*Ksp*^*Cre*^ mice. **D** Representative PI staining images showing cisplatin-induced PTCs cell death. Scale bar = 100 μm. **E** Quantification of cell death using PI/Hoechst staining and Image J software. *n* = 6, ns not significant, ****P < 0.0001. **F** After 24 h of cisplatin treatment, cells were stained with Mitotracker and MitoSOX probes. Scale bar = 10 μm. **G** ROS production in *Idh1*^*WT/WT*^ and *Idh1*^*WT/Mut*^ PTCs induced by cisplatin was detected using DCFH-DA and C11-BODIPY probes, followed by analysis with flow cytometry. **H** Measurement of mitochondrial membrane potential (MMP) in *IDH1*^*WT/WT*^ and *IDH1*^*WT/Mut*^ cells after cisplatin treatment using JC-10 staining method. Results showed that the IDH1-R132H mutation exacerbated cisplatin-induced reduction in PTCs MMP. **I** Quantification of the ratio of red aggregates to green monomers. Scale bar = 10 μm.

To gain deeper insight into the exacerbating effects of the IDH1-R132H mutation on cisplatin-induced renal tubular cell ferroptosis and oxidative stress, we conducted further using stable cell lines (mouse renal tubular cells, TECs) expressing Flag-IDH1-WT and Flag-IDH1-R132H proteins (Fig. 3A). Colony formation assays indicated that colonies formed by the *IDH1*^*WT/Mut*^ group were smaller than those of the *IDH1*^*WT/WT*^ group, suggesting that the IDH1-R132H mutation significantly inhibits tubular epithelial cell proliferation (Fig. 3B). Furthermore, CCK8 assays demonstrated significantly reduced cell viability in the *IDH1*^*WT/Mut*^ group compared to the *IDH1*^*WT/WT*^ group (Fig. 3C). We further isolated primary PTCs from the *Idh1*^*WT/Mut*^*Ksp*^*Cre*^ *mice* underwent extensive cell death following cisplatin treatment, exhibiting morphological characteristics consistent with ferroptosis. Pretreatment with two lipid ROS scavengers, Fer-1 and Lip-1, but not zVAD, significantly inhibited cisplatin-induced cell death (Fig. 3D, E, S2). Expression analysis of oxidative stress and ferroptosis-related proteins in *vitro* cultured PTCs yielded results consistent with those observed in vivo (Fig. S3).

Prior studies have indicated that mitochondrial dysfunction plays a crucial role in the pathogenesis of Cis-AKI [20]. To examined whether the IDH1-R132H mutation affects cell death via the mitochondrial pathway, we observed changes in mitochondrial morphology, mitochondrial ROS accumulation, and MMP in PTCs isolated from *Idh1*^*WT/WT*^*Ksp*^*Cre*^ and *Idh1*^*WT/Mut*^*Ksp*^*Cre*^ following cisplatin treatment. The results revealed that compared to the *Idh1*^*WT/WT*^ group, the IDH1-R132H mutation significantly exacerbated cisplatin-induced disruption of mitochondrial morphology in PTCs, leading to increased mitochondrial fragmentation (Fig. 3F). Additionally, flow cytometry analysis using oxidative stress and lipid peroxidation-related markers MitoSOX, DCFH-DA, and C11-BODIPY detected widespread distribution of mitochondrial ROS within fragmented mitochondria in the *Idh1*^*WT/Mut*^ group, indicating aggravated oxidative stress damage within the mitochondria (Fig. 3G). Furthermore, compared to the *Idh1*^*WT/WT*^ group, a reduction in MMP of PTCs was observed following cisplatin treatment, with a more pronounced decrease in the *Idh1*^*WT/Mut*^ group (Fig. 3H, I). These findings suggest that the IDH1-R132H mutation significantly promotes the accumulation of mitochondrial ROS in cisplatin-induced PTCs, exacerbating intracellular oxidative stress damage and increasing the susceptibility of PTCs to cisplatin-induced ferroptosis.

### IDH1-R132H mutation inhibited the expression of *Ndufa1* by upregulating the methylation level of *Ndufa1* promoter

**Fig. 4:**
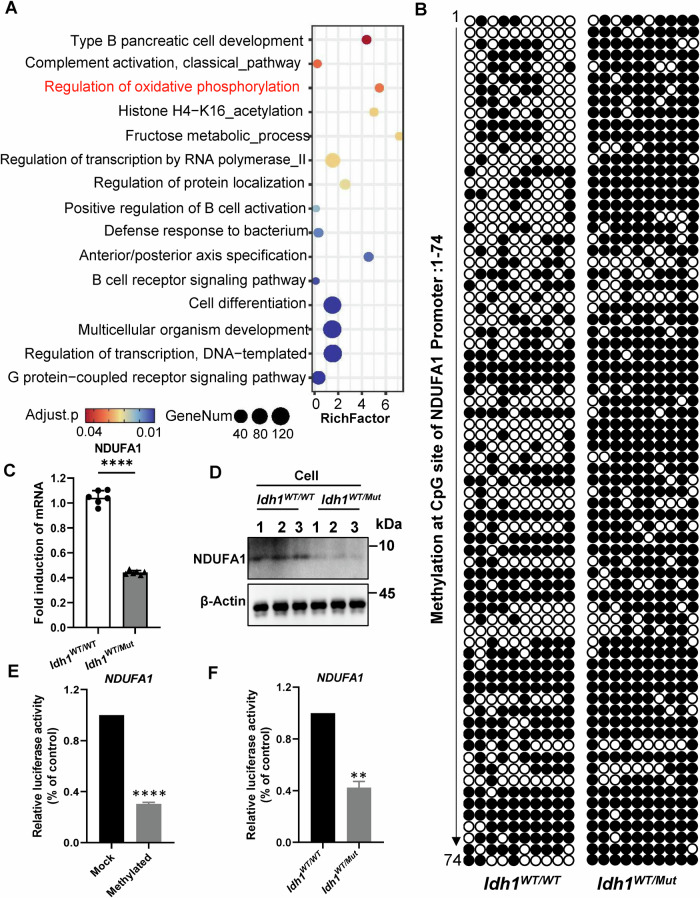
IDH1-R132H mutation significantly enhanced the methylation modification level of Ndufa1 promoter. **A** GO enrichment analysis was performed on genes in the differentially upregulated promoter region in RBBS sequencing, and the most significantly enriched biological process items were selected for display. **B** Clones of the BSP products were analyzed by DNA sequencing. 10 clones for each amplicon were sequenced; each row represents the methylation status of an individual sequenced clone. Each circle represents one CpG site, with ● depicting methylated site and ○ depicting unmethylated sites. **C**, **D** RT-PCR quantification and WB of NDUFA1 expression relative to β-actin in PTCs isolated from *Idh1*^*WT/WT*^*Ksp*^*Cre*^ and *Idh1*^*WT/Mut*^*Ksp*^*Cre*^ mice. *n* = 6, ****P < 0.0001. **E** The combination of MSssI treatment and luciferase activity was used to determine whether CpG methylation directly contributes to transcriptional repression of the *Ndufa1* gene. Compared to control cells (fragments not treated with MSssI), luciferase activity was significantly downregulated in 293 T cells containing the *Ndufa1* promoter reporter construct treated with MsssI, ****P < 0.0001. **F** When the IDH-R132H overexpression plasmid or empty vector was co-transfected with pGL4-Ndufa1 and renilla luciferase vector into 293 T cells, luciferase activity of *Idh1*^*WT/ Mut*^ group was reduced as compared to the *Idh1*^*WT /WT*^group, **P < 0.01.

The IDH1-R132H mutation primarily affects cellular functions by altering epigenetic regulatory mechanisms. We hypothesized that the IDH1-R132H mutation in renal tubules might alter the methylation patterns of mitochondrial-related genes, thereby exacerbating cisplatin-induced kidney injury. RRBS revealed 15,296 upregulated and 7,947 downregulated differentially methylated genes in the exonic regions of the kidneys of *Idh1*^*WT/Mut*^*Ksp*^*Cre*^ mice, with 12,935 upregulated and 4,409 downregulated differentially methylated genes observed in the promoter regions (using *Idh1*^*WT/WT*^*Ksp*^*Cre*^ mice as the control group) (Fig. S4). Gene enrichment analysis indicated activation of signals related to histone acetylation, cellular metabolism, and oxidative phosphorylation in the promoter regions (Fig. 4A). Among the upregulated methylation sites, significantly enhanced methylation was observed in the promoter region of *Ndufa1*, a gene associated with mitochondrial respiratory chain assembly. Further bisulfite sequencing PCR (BSP) yielded results consistent with RRBS sequencing. The differentially methylated region of the *Ndufa1* promoter was located in the chrX:37191032-37191910 region, containing 74 CpG sites. In *vitro* experiments, approximately 47.4% of CpG sites in this segment of *Ndufa1* were methylated in the *Idh1*^*WT/WT*^ group, while in *Idh1*^*WT/Mut*^ group, approximately 82.4% of CpG sites were methylated (Fig. 4B, Fig. S5A). Approximately 54% of CpG sites in this segment of *Ndufa1* were methylated in the kidney tissue of the *Idh1*^*WT/WT*^*Ksp*^*Cre*^ mice, while in *Idh1*^*WT/Mut*^*Ksp*^*Cre*^ group, approximately 80% of CpG sites were methylated (Fig. S6A). QT-PCR and western blot analyses of kidney tissue and PTCs in *vitro* indicated a decrease in the expression levels of NDUFA1 (Fig. 4C, D and S6B, S6C).

To test whether CpG methylation plays a direct role in transcriptional repression of the *Ndufa1* gene, we performed patch-methylation using MSssI in combination with luciferase activity assays. Successful methylation was identification by methylation-sensitive restriction endonucleases SmaI I. Luciferase activities were significantly downregulated in 293 T cells transfected with reporter vector containing *Ndufa1* promoter treated with MSssI as compared to control cells (fragments not treated with MSssI) (Fig. 4E). When the IDH-R132H overexpression plasmid or empty vector was co-transfected with pGL4-Ndufa1 and renilla luciferase vector into 293 T cells, luciferase activity of *IDH1*^*WT/Mut*^ group was reduced as compared to the *IDH1*^*WT/WT*^ group (Fig. 4F). These findings suggest that CpG methylation suppresses *Ndufa1* gene promoter activity and plays a direct role in silencing *Ndufa1*. That this methylation at CpG sites is regulated by IDH1-R132H. These results underscore the transcriptional and translational suppression of *Ndufa1* due to the upregulation of methylation levels caused by the IDH1-R132H mutation.

### NDUFA1 involved in cisplatin-induced mitochondrial dysfunction and oxidative stress in IDH1-R132H mutant tubular epithelial cells

**Fig. 5:**
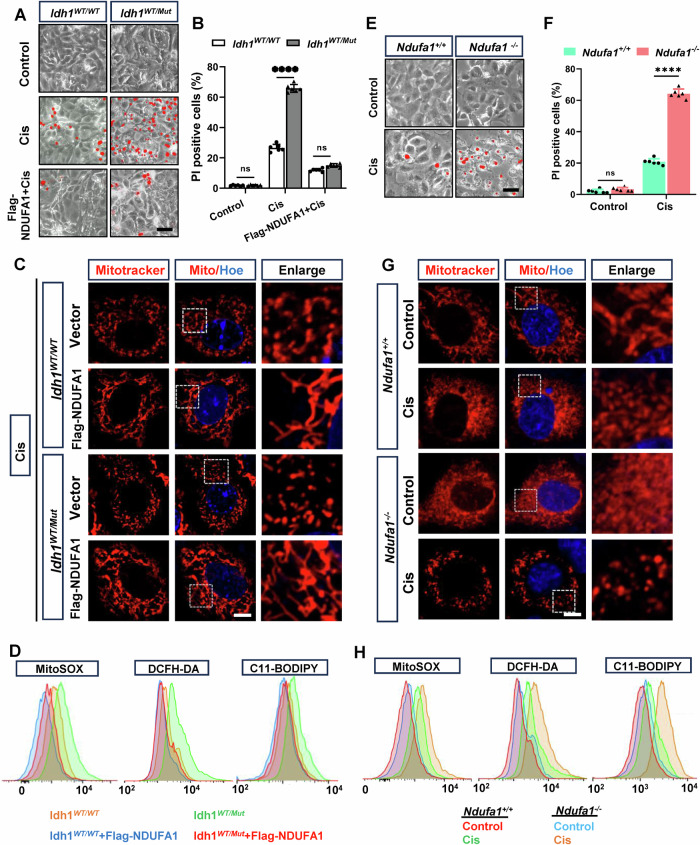
NDUFA1 involvement in cisplatin-induced mitochondrial dysfunction and oxidative stress in tubular epithelial cell with IDH1-R132H mutation. **A** Representative PI staining images depicting cisplatin-induced PTCs cell death isolated from *Idh1*^*WT/WT*^*Ksp*^*Cre*^ and *Idh1*^*WT/Mut*^*Ksp*^*Cre*^ mice. Scale bar = 100 μm. **B** Quantification of cell death using PI/Hoechst staining and Image J software. **C** Overexpression of NDUFA1 alleviates cisplatin-induced mitochondrial dysfunction in renal tubular epithelial cells with IDH1-R132H mutation, as observed by Mito-tracker staining for mitochondrial morphology following cisplatin stimulation. Scale bar = 10 μm. Nuclei were counterstained with Hoechst. **D** MitoROS generation, ROS production and lipid ROS level of *Idh1*^*WT/WT*^ and *Idh1*^*WT/Mut*^ PTCs infected with a lentivirus encoding nothing (vector) or Flag-NDUFA1 was measured using MitoSOX, DCFH-DA and C11-BODIPY probe separately analyzed by FACS. **E** Representative PI staining images showing *Ndufa1*^*+/+*^ and *Ndufa1*^*−/−*^ HK-2 cell death induced by cisplatin. Scale bar = 100 μm. **F** Quantification of cell death for using PI/Hoechst staining and Image J software. **G** Deletion of *Ndufa1* exacerbates cisplatin-induced mitochondrial dysfunction in renal tubular epithelial cells, as observed by Mito-tracker staining for mitochondrial morphology following cisplatin stimulation. Scale bar = 10 μm. Nuclei were counterstained with Hoechst. **H** Deletion of *Ndufa1* exacerbates cisplatin-induced renal tubular injury, as detected by MitoROS generation, ROS generation and lipid ROS levels using MitoSOX, DCFH-DA and C11-BODIPY probes in *Ndufa1*^*+/+*^ and *Ndufa1*^*−/−*^ tubular cells.

To elucidate the role of NDUFA1 in the cisplatin-induced death of tubular epithelial cell with the IDH1-R132H mutation, we overexpressed NDUFA1 in PTCs from *Idh1*^*WT/WT*^*Ksp*^*Cre*^ and *Idh1*^*WT/Mut*^*Ksp*^*Cre*^ mice and treated them with 25 μM cisplatin for 24 h (Fig. S7A). Our results demonstrated that overexpression of NDUFA1 significantly mitigated cisplatin-induced cell death in PTCs with the IDH1-R132H mutation, as evidenced by a marked reduction in PI-positive cells (Fig. 5A, B). Furthermore, these cells exhibited improved MMP and decreased mitochondrial ROS accumulation (Fig. 5C, D). Consistently, *Ndufa1* knockout (*Ndufa1*^*−/−*^) exacerbated cisplatin-induced cell death in HK-2 cells, indicated by an increased number of PI-positive cells following cisplatin treatment compared to wild-type controls (*Ndufa1*^*+/+*^) (Fig. 5E, F and S8A, B). Additionally, mitochondrial dysfunction was more pronounced in *Ndufa1*^*−/−*^ HK-2 cells, characterized by a significant reduction in MMP, increased ROS accumulation, and elevated lipid peroxidation levels, as evidenced by higher DCFH-DA and C11-BODIPY fluorescence intensities (Fig. 5G, H). These findings indicate that NDUFA1 plays a critical role in protecting against cisplatin-induced mitochondrial dysfunction and oxidative stress in tubular epithelial cell harboring the IDH1-R132H mutation. By maintaining mitochondrial integrity and reducing oxidative damage, NDUFA1 overexpression provides a protective effect against cisplatin-induced cell death in these cells.

### NDUFA1 inhibited mitochondrial oxidative stress by interacting with FSP1

**Fig. 6:**
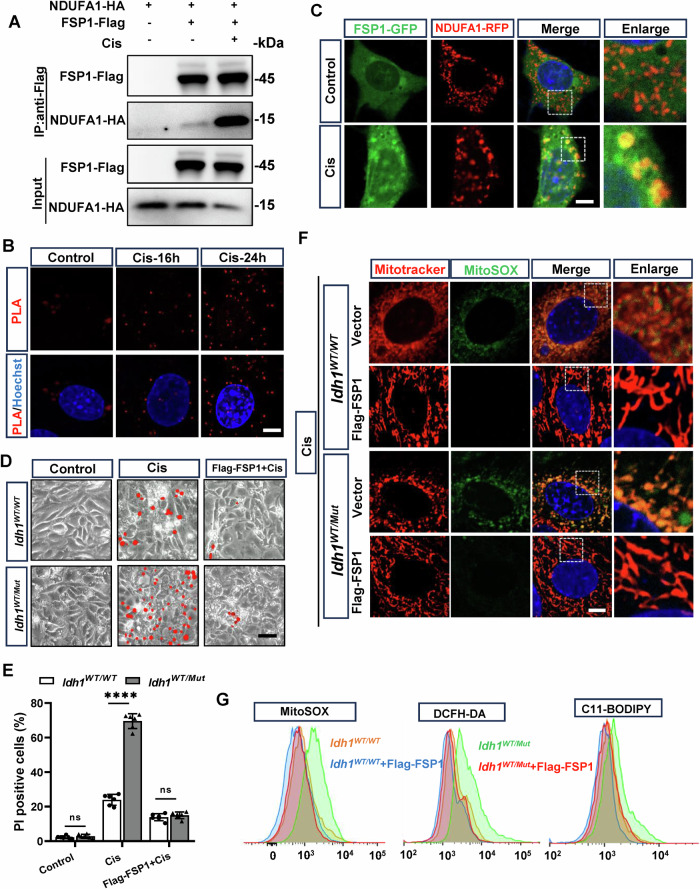
NDUFA1 regulates cisplatin-induced renal tubular epithelial cell injury through modulation of FSP1. **A** Immunoprecipitation confirmed the direct interaction between NDUFA1 and FSP1. HEK293T cells were co-transfected with Flag-FSP1 and HA-NDUFA1, followed by immunoprecipitation using anti-Flag M2 beads to pull down Flag-FSP1. The results demonstrated a direct interaction between NDUFA1 and FSP1. **B** Proximity ligation assay (PLA) detected the physical interaction between FSP1 and NDUFA1 in tubular epithelial cells after 24 h of cisplatin treatment. Red dots indicate positive PLA signals. Nuclei were counterstained with Hoechst. Scale bar = 10 μm. **C** Confocal laser scanning microscopy was used to observe the co-localization of FSP1 and NDUFA1 in tubular epithelial cells after 24 h of cisplatin treatment. Nuclei were stained with Hoechst for visualization. Scale bar = 10 μm. **D** PTCs were isolated from *Idh1*^*WT/WT*^*Ksp*^*Cre*^ and *Idh1*^*WT/Mut*^*Ksp*^*Cre*^ mice. After PTCs infected with empty vector or Flag-Fsp1 lentivirus. 25 μm cisplatin were added. Representative PI staining images indicated cisplatin-induced PTC death. Scale bar = 100 μm. **E** Quantification of cell death using PI/Hoechst staining and Image J software. **F** PTCs were stained with Mitotracker and MitoSOX probes. Scale bar = 10 μm. **G** ROS generation and lipid ROS levels were detected using MitoSOX, DCFH-DA and C11-BODIPY probes in PTCs.

Previous studies have suggested that both NDUFA1 and FSP1 play critical roles in regulating the NADH-dependent CoQ oxidoreductase system [21]. Immunoprecipitation assays and proximity ligation assays confirmed a direct interaction between NDUFA1 and FSP1 (Fig. 6A, B). Furthermore, we observed a significant increase in the co-localization of NDUFA1 and FSP1 within mitochondria following cisplatin treatment (Fig. 6C).

**Fig. 7:**
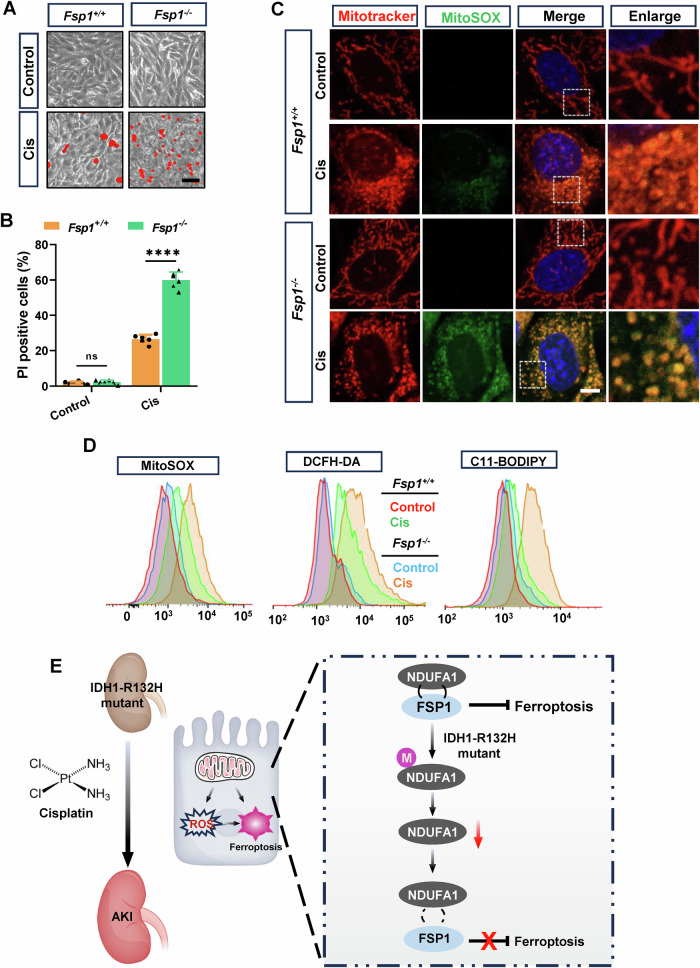
*Fsp1* deficiency enhances cisplatin-induced ferroptosis and oxidative stress in tubular epithelial cells. **A** Representative PI staining images showing *Fsp1*^*+/+*^ and *Fsp1*^*−/−*^ mouse tubular epithelial cell (TEC) death induced by cisplatin. Scale bar = 100 μm. **B** Quantification of cell death for using PI/Hoechst staining and Image J software. **C** Deletion of *Fsp1* exacerbates cisplatin-induced mitochondrial dysfunction in tubular epithelial cells, as observed by Mito-tracker staining for mitochondrial morphology following cisplatin stimulation. *n* = 6, ns = not significant, ****P < 0.0001. Scale bar = 10 μm. **D** Deletion of *Fsp1* exacerbates cisplatin-induced renal tubular injury. ROS generation and lipid ROS levels were detected using MitoSox, DCFH-DA and C11-BODIPY probes in *Fsp1*^*+/+*^ and *Fsp1*^*−/−*^ cells. **E** Schematic diagram of the mechanism by which IDH1R132H exacerbates Cis-AKI.

To further elucidate the role of FSP1 in the cisplatin-induced death of tubular epithelial cell with the IDH1-R132H mutation, we overexpressed FSP1 in PTCs from mice (Fig. S7B). The overexpression of FSP1 led to a marked reduction in cisplatin-induced cell death in PTCs with the IDH1-R132H mutation, as shown by the decreased number of PI-positive cells (Fig. 6D, E). This protective effect was also accompanied by a significant reduction in mitochondrial ROS accumulation and lipid peroxidation levels, evidenced by lower staining intensities for oxidative stress markers (Fig. 6F, G). Consistently, FSP1 knockout (*Fsp1*^*−/−*^) exacerbated cisplatin-induced cell death in TECs, indicated by an increased number of PI-positive cells and more pronounced mitochondrial dysfunction (Fig. 7A, B and S8C, D), with significant increases in mitochondrial ROS accumulation and lipid peroxidation levels (Fig. 7C). Flow cytometry analysis further supported these observations, showing higher levels of MitoSOX, DCFH-DA, and C11-BODIPY fluorescence in *Fsp1*^*−/*−^ cells compared to *Fsp1*^*+/+*^ cells under cisplatin treatment (Fig. 7D). These findings suggest that NDUFA1 regulates cisplatin-induced tubular epithelial cells injury through FSP1 mediation in the context of the IDH1-R132H mutation. The direct interaction and mitochondrial co-localization of NDUFA1 and FSP1 underscore the significance of this pathway in mitigating cisplatin-induced oxidative stress and ferroptosis. Targeting the interactions between NDUFA1 and FSP1 could, therefore, represent a promising therapeutic strategy to protect against cisplatin-induced nephrotoxicity, especially in the presence of IDH1-R132H mutations.

## Discussion

As the primary organs for metabolizing and excreting cisplatin, the kidneys are highly vulnerable to its severe side effects [22]. In this study, we observed that a common mutation in the gene IDH1, occurring within the citric acid cycle, exacerbates Cis-AKI, characterized by more severe tubular injury, necrosis, dilation, and interstitial inflammation. This mutation also increases renal lipid peroxidation levels and sensitizes the kidneys to cisplatin-induced ferroptosis. Furthermore, the IDH1-R132H mutation affects the Keap1/Nrf2/HO-1 signaling pathway, rendering the kidneys more susceptible to oxidative stress. Further investigation revealed that the mutation induces mitochondrial dysfunction, leading to the accumulation of mitochondrial ROS within the cells, thereby increasing intracellular ROS and lipid peroxidation levels, and exacerbating oxidative stress damage. Additionally, the mutation inhibits the proliferative capacity of tubular epithelial cells and alters the expression levels of key genes in the oxidative stress pathway, ultimately resulting in cell death. Mechanistic studies further demonstrated that the IDH1-R132H mutation increases the methylation levels of the *Ndufa1* promoter, inhibiting the transcription and expression of *Ndufa1*, thus causing mitochondrial dysfunction. The interaction between NDUFA1 and FSP1 aids in clearing mitochondrial ROS, alleviating oxidative stress; however, the IDH1-R132H mutation inhibits NDUFA1 expression, affecting its interaction with FSP1, resulting in the accumulation of ROS and lipid peroxidation, and aggravating oxidative damage and cell death (Fig. 7E). Overall, these findings highlight the critical role of the IDH1-R132H mutation in cisplatin-induced kidney injury, providing important insights for the development of personalized treatment strategies. Understanding these mechanisms is also crucial for gaining deeper insights into the injuries that cisplatin causes to other organs.

Under physiological conditions, the body generates oxygen radicals through both enzymatic and non-enzymatic systems. These radicals act on polyunsaturated fatty acids in cell membranes, triggering lipid peroxidation reactions. Excessive lipid peroxidation products exhibit cytotoxicity [23]. ROS are key triggers for lipid peroxidation, and studies show that cisplatin promotes ROS generation through multiple mechanisms. Cisplatin directly targets mitochondria, disrupting the electron transport chain (ETC), particularly complexes I and III, leading to electron leakage and the formation of superoxide anions. These are then converted into reactive oxidative mediators like hydrogen peroxide, constituting a primary pathway for cisplatin-induced ROS production [24, 25]. Additionally, cisplatin catalyzes the release of iron-sulfur (Fe-S) clusters from the ETC, further disrupting electron transport and increasing ROS generation. The released iron ions participate in the Fenton reaction, producing highly reactive hydroxyl radicals and exacerbating oxidative stress [26, 27]. Cisplatin also indirectly raises ROS levels by inhibiting the cellular antioxidant system, suppressing glutathione (GSH) synthesis and regeneration, reducing catalase and peroxidase activities, and diminishing ROS clearance [28, 29]. In some cells, cisplatin activates NADPH oxidase, further elevating ROS levels both at the cell surface and within the cells [25, 30]. Our study indicates that the IDH1-R132H mutation intensifies oxidative stress and significantly raises the levels of 4-HNE and MDA in kidneys treated with cisplatin. This leads to an increased accumulation of ROS and lipid peroxidation products, making tubular epithelial cell more susceptible to ferroptosis. Ferroptosis is an iron-dependent, non-apoptotic form of programmed cell death characterized by iron accumulation and increased lipid peroxidation, resulting in membrane disruption and eventual cell death [31–34]. Previous studies have demonstrated the involvement of ferroptosis in Cis- AKI. In this study, we found that the levels of ACSL4 upregulation in the kidneys of *Idh1*^*WT/WT*^ and *Idh1*^*WT/Mut*^ mice were similar, while the expression of GPX4 and SLC7A11 was downregulated to the same extent, and FSP1 showed no significant changes. The Keap1/Nrf2/HO-1 signaling pathway can inhibit ferroptosis and alleviate cisplatin-induced acute kidney injury [21, 35, 36]. Under oxidative stress, cysteine residues on Keap1 are modified, leading to conformational changes that prevent effective promotion of Nrf2 degradation [37, 38]. Nrf2 stabilizes and accumulates in the cell, translocating to the nucleus to induce the expression of various antioxidant and detoxification enzymes, including HO-1, thereby enhancing the cell’s resistance to oxidative stress [39]. The IDH1-R132H mutation leads to reduced Nrf2 activity, weakening the cell’s defense against oxidative stress, making the kidneys more susceptible to damage [40]. Similarly, considering the high oxygen environment in the lungs, this could also be a potential mechanism by which the IDH1-R132H mutation exacerbates cisplatin-induced lung toxicity. By disrupting the redox balance in the lung microenvironment, the mutation may impair the Keap1/Nrf2/HO-1 antioxidant pathway, reducing the antioxidant capacity of lung tissue and making lung cells more susceptible to oxidative stress-induced damage [41]. This hypothesis requires further experimental validation. Oxidative stress is a state of cellular and tissue damage caused by excessive ROS production or insufficient antioxidant defenses [42]. Previous studies have shown that mitochondria are one of the main sources of ROS within cells [43]. Mitochondrial dysfunction leads to abnormal cellular energy metabolism, impaired cell signaling, and increased intracellular ROS levels, which exceed the cell’s clearance capacity, making cells more susceptible to oxidative stress and ferroptosis [44–46].

Both IDH1 and IDH2 are integral to the tricarboxylic acid (TCA) cycle, serving as pivotal enzymes within its cascade. The IDH1-R132H mutation confers upon the enzyme a neomorphic activity, facilitating the conversion of α-ketoglutarate (α-KG) into D-2-hydroxyglutarate (D-2HG) [12, 47]. Given the structural resemblance between D-2HG and α-KG, D-2HG can competitively impede α-KG-dependent hydroxylases, predominantly involved in DNA, RNA, and histone demethylation. Consequently, this inhibition leads to epigenetic alterations, metabolic reprogramming, and mitochondrial dysfunction. Research suggests that the IDH1-R132H mutation influences the activity of methylation-related enzymes engaged in DNA/RNA/histone modifications, culminating in intricate genetic and epigenetic changes [48, 49]. For example, ten-eleven translocation (TET) dioxygenases, reliant on α-KG and Fe2^+^, play a pivotal role in DNA demethylation by converting 5-methylcytosine to 5-hydroxymethylcytosine. With D-2HG competitively binding to the active site of TET2, its demethylation function is hindered, resulting in elevated DNA methylation levels within cells [50]. These alterations in DNA methylation status may precipitate transcriptional repression and gene expression downregulation.

Our investigation further delineates the impact of the IDH1-R132H mutation on the methylation status of *Ndufa1*, a gene pivotal in regulating mitochondrial function. *Ndufa1* encodes a crucial component of mitochondrial complex I, facilitating the transfer of electrons from NADH to coenzyme Q, thereby playing an essential role in the activity of the mitochondrial respiratory chain complex I [51, 52]. Notably, studies indicate that ROS primarily emanate from electron leakage in mitochondrial electron transport chain complex I. Inhibition of *Ndufa1* expression directly influences mitochondrial function, encompassing mitochondrial membrane potential, complex activity, ATP generation, and ROS production [53]. Moreover, the IDH1 mutation can deplete NAD(P)H, disrupting the antioxidant system and impeding the reduction of oxidized glutathione (GSSG) to reduced GSH within mitochondria, thereby fostering ROS accumulation [54]. Tubular epithelial cells are rich in mitochondria, providing the necessary energy to maintain their normal function. The abundance of mitochondria makes the kidneys more vulnerable to the impacts of mitochondrial dysfunction [55]. Similarly, given the liver’s active metabolic state, cisplatin’s hepatotoxic effects may be exacerbated by the IDH1-R132H mutation. This mutation inhibits the production of NAD(P)H, reduces the supply of the antioxidant GSH, and impairs the oxidative defense capacity of liver cells. The resulting excessive production of ROS and reactive nitrogen species (RNS) leads to lipid peroxidation, DNA damage, and hepatocyte necrosis, which warrants further experimental validation [56]. Under cisplatin intervention, FSP1 relocates to mitochondria, where NDUFA1 augments its interaction with mitochondrial membrane-bound FSP1, consequently clearing mitochondrial ROS and mitigating mitochondrial oxidative stress. Phenotypic experiments involving *FSP1* overexpression and knockout in tubular epithelial cells underscore the exacerbation of oxidative stress upon *Fsp1* knockout under cisplatin intervention, while *FSP1* overexpression significantly attenuates cisplatin-induced tubular damage. Interestingly, FSP1 knockout resulted in more severe injury in the Cis-AKI model compared to the IRI-AKI model, likely due to the distinct nature of these models [57, 58]. Ischemia-reperfusion injury is driven by physical hypoxia and the inflammatory response upon reperfusion, affecting multiple cell types and activating various signaling pathways, which may overshadow the role of FSP1. In contrast, cisplatin directly targets renal tubular epithelial cells, significantly triggering ferroptosis and disrupting FSP1-mediated defense mechanisms [25]. Studies elucidate that FSP1 can reduce ubiquinone (CoQ) to the antioxidant ubiquinol (CoQH2) in the cell membrane, thus impeding lipid peroxidation reactions and inhibiting ferroptosis [59, 60]. Furthermore, through co-immunoprecipitation, immunofluorescence, and PLA techniques, we have unraveled the significant enhancement of the interaction between NDUFA1 and FSP1 following cisplatin intervention. Featuring a short N-terminal hydrophobic sequence, FSP1 mediates its localization to mitochondria and interaction with NDUFA1 via the N-terminal mitochondrial signal peptide. Functionally, FSP1 is capable of reducing CoQ to CoQH2. CoQH2, being a lipophilic free radical scavenging antioxidant, effectively clears ROS within mitochondria during mitochondrial oxidative damage, thereby mitigating the release and leakage of ROS from mitochondria, consequently preventing lipid peroxidation and inhibiting ferroptosis [21, 61, 62].

In summary, this study reveals that the IDH1-R132H mutation enhances NDUFA1 promoter methylation, downregulates its transcription and expression, inhibits the interaction between NDUFA1 and FSP1, thereby affecting FSP1 localization in mitochondria and its ability to reduce CoQ to CoQH2, leading to accumulation of ROS and lipid peroxides, thereby exacerbating cisplatin-induced oxidative damage and ferroptosis in tubular epithelial cells. Overall, these findings highlight the critical role of the IDH1-R132H mutation in cisplatin-induced renal injury, providing important insights for developing personalized treatment strategies for cancer patients.

## Methods

### Mice

Ksp-Cre mice were generously provided by Professor Hui-Yao Lan from The Chinese University of Hong Kong [63]. IDH1-LSL mice were sourced from Professor Tak Wah Mak at the University of Toronto. Cre-mediated excision of LSL allows expression of the IDH1(R132H) protein [64]. Male mice (10- to 12-week-old, 25–28 g body weight) were used in this study. A detailed description of their genetic background were provided in Supplementary Methods and Fig. S9.

### Establishment of cisplatin-induced AKI mouse model

*Idh1*^*WT/WT*^*Ksp*^*Cre*^ mice and IDH1-R132H mutant (*Idh1*^*WT/Mut*^*)×Ksp*^*Cre*^ mice were housed in a pathogen-free environment and randomly divided into the following groups (*n* = 6 per group): control group, cisplatin group, cisplatin + Liproxstatin-1 (Lip-1) group, and cisplatin + benzyloxycarbonyl-Val-Ala-Asp(OMe)-fluoromethylketone (zVAD) group. All mice were male, weight with 25–28 g. In the cisplatin group, mice were administered a single intraperitoneal injection of cisplatin (HY-17394, MedChemExpress, USA) at a dose of 10 mg/kg. Lip-1 (HY-12726, MedChemExpress, USA) was dissolved in DMSO for storage and was adjusted to 2% DMSO with saline (1 mg/ml) before injection. In the cisplatin + Lip-1 group, Lip-1 was administered via intraperitoneal injection at a dose of 10 mg/kg both 1 day and 2 h prior to cisplatin injection, followed by a single intraperitoneal injection of cisplatin at a dose of 10 mg/kg. In the cisplatin + zVAD group, zVAD (HY-16658B, MedChemExpress, USA) was administered via intraperitoneal injection at a dose of 5 mg/kg 1 h prior to cisplatin injection. After handling the mice, the investigators were blinded when assessing the outcome.

### Cell viability and cell death assessment

Detailed methods were provided in Supplementary information.

### Analysis of intracellular reactive oxygen species (ROS) and mitochondrial ROS

For intracellular ROS detection, the cells were subjected to incubation with dichloro-dihydro- fluorescein diacetate (DCFH-DA, S0033S, Beyotime) at a final concentration of 10 μM, in FBS-free DMEM, at 37 °C for 30 min in the absence of light. For mitochondrial ROS, cells were stained with Mito-tracker and MitoSoxTM (M36009, ThermoFisher). Subsequently, they underwent two PBS washes. Following the manufacturer’s instructions, the fluorescence signal was quantified by the confocal ZEISS LSM800 microscope or CytoFLEX cytometer instrument from Beckman Coulter.

### Measurement of lipid peroxidation, mitochondrial morphology and mitochondrial membrane potential (MMP)

Detailed methods were provided in Supplementary information.

### Duolink proximity ligation assay (PLA)

PLA with the Duolink system (Sigma-Aldrich) were performed. Cells were seeded, treated with cisplatin, fixed with paraformaldehyde, and permeabilized with Triton X-100. After blocking, cells were incubated with primary antibodies (1:200) overnight at 4 °C. They were washed with buffer A, then incubated with anti-rabbit PLUS and anti-mouse MINUS PLA probes for 1 h. Ligation and amplification were done with Duolink reagents at 37 °C. Cells were washed with buffer B, counterstained with DAPI, rinsed, and imaged using a ZEISS LSM800 confocal microscope. Detailed methods were provided in Supplementary information.

### Immunostaining and imaging, histologic analysis of kidney sections, Western blot analysis

Detailed methods and antibodies were provided in Supplementary information. Uncropped blots are shown in Supplementary material file.

### Isolation and culture of primary renal tubular epithelial cells (PTCs)

Primary renal tubular epithelial cells (PTCs) from *Idh1*^*WT/WT*^*Ksp*^*Cre*^ mice and *Idh1*^*WT/Mut*^*Ksp*^*Cre*^ were freshly isolated. Cortical tissue was dissected, digested with 0.75 mg/ml collagenase at 37 °C for 30 min, and digestion was terminated using DMEM/F12 containing 10% FBS. The digested tissue was filtered through a 100 μM sieve and suspended in HBSS. Pure PTCs were isolated using the Percoll gradient centrifugation method. Finally, freshly isolated PTCs were collected. The cells were seeded onto collagen-coated culture dishes and cultured in DMEM-F12. After 5–6 days, cultured PTCs were used for subsequent experiments. In all cell experiments, when cell confluence reached 80%, cisplatin was administered at a concentration of 25 μM at four time points: 0 h, 6 h, 12 h, and 24 h.

### Ligase Independent Clone (LIC)

In LIC experiments, Exonuclease III digests vector and DNA fragment ends to expose 15 bp homologous arms. E. coli then joins complementary DNA fragments. The reaction mix includes pBoB-N-Flag or pBoB-C-Flag vector (15–50 ng), target fragment (50–100 ng), 10X Exo III buffer (1 μl), and ddH2O to 10 μl. After cooling on ice for 10 min, Exonuclease III (1 μl, 20U) is added and incubated on ice for 60 min. The reaction is stopped with 1 μl of 0.5 M EDTA, heated at 60 °C for 5 min, and cooled on ice. Detailed methods were provided in Supplementary information.

### Bisμlfite sequencing PCR (BSP)

Detailed methods were provided in Supplementary information.

### Simplified Reduced Representation Bisulfite Sequencing (RRBS) Library preparation, sequencing and analysis

Detailed methods were provided in Supplementary information.

### Real‑time quantitative polymerase chain reaction and Co-immunoprecipitation

Detailed methods and antibodies were provided in Supplementary information.

### Generation of knockout cell lines using the CRISPR-Cas9 technique

*Ndufa1* and *Fsp1* were knocked out in cell lines using CRISPR-Cas9. Guide RNAs targeting *Ndufa1* (5′-CTCCCCGGACTCTCCGTCAT-3′) and *Fsp1* (5′-TGCACGTGGTGATCGTGGGC-3′) were cloned into lentiGuide vectors. Lentivirus collected 48 h post-transfection infected human proximal tubule epithelial cells (HK-2) or mouse tubular epithelial cells (TECs) [65] with 10 μg/ml polybrene. Cells were selected with puromycin for 3 days, then sorted into 96-well plates and cultured for 3–4 weeks. Colonies were confirmed by sequencing and western blot. Detailed methods were provided in Supplementary information.

### Patch methylation and identification

A 878 bp fragment containing the mouse Ndufa1 promoter (chrX:37191032-37191910) was cloned into pGL4 luciferase reporter vector (Promega, USA). The regions of interest were then excised using the restriction endonucleases KpnI and Hind III (NEB, USA), gel purified, and methylated with SssI recombinant methyltransferas and S-adenosylmethionine (ZYMO RESEARCH, USA) according to the recommendations provided by the manufacturer. Methylated fragments were purified by gel extraction and ligated back into the pGL4 vector. Methylation was identification by digesting with the methylation-sensitive restriction endonucleases SmaI (ThermoFisher). Controls included mockmethylated constructs similarly generated but omitting the SssI.

### Luciferase reporter gene assay

The methylated or mock-methylated pGL4-Ndufa1 constructs were then Co-transfected with renilla luciferase vector into 293 T cells via calcium phosphate precipitation. 48 h later, The cells were suspended in 100 ul of reporter lysis buffer (Yeasen, China), and lysed by freezing and thawing, while luciferase assays were performed using the Dual-Luciferase Reporter Assay Kit (Yeasen, China), measuring fluorescence by SpectraMax i3x. All data were normalized to Renilla luciferase luminescence derived from the co-transfected pRL-SV40 vector.

### Plasmid construction, Lentivirus preparation and infection

The IDH1, IDH1-R132H, NDUFA1 and FSP1, were generated through a two-round PCR amplification procedure using specific primers. Subsequently, all of these DNA fragments were cloned into the BamHI and XhoI sites of the modified lentiviral vector pBOB, which lacked a tag or included Flag/HA/GFP/RFP tags. The Exo III-assisted ligation- independent cloning method was employed for subcloning purposes. To validate the constructed plasmids, we underwent thorough verification via DNA sequencing. Detailed methods of lentivirus preparation and infection were provided in Supplementary information.

### Statistical analyses

Results were represented at least three independently performed experiments. Statistical analysis was performed with Prism software (GraphPad Software, Inc.). The data are expressed as mean ± SD. Group comparisons were conducted using an unpaired *t*-test, and for multiple comparisons, a one-way ANOVA was employed, followed by post hoc Bonferroni correction. Statistical significance was attributed to differences with *p* < 0.05.

## Supplementary information

Supplementary Materials

Supplementary original western blots gel

## Data Availability

The datasets used in the current study are available from the corresponding author (YX) upon reasonable request. The reduced representation bisulfite sequencing data has been deposited in the National Center for Biotechnology Information Sequence Read Archive (SRA) database, https://www.ncbi.nlm.nih.gov/sra (accession no. PRJNA1160432). The original western blot data are provided in Supplementary Materials (Original western blots gel).

## References

[1] Duan M, Leng S, Mao P. Cisplatin in the era of PARP inhibitors and immunotherapy. Pharm Ther. 2024;258:108642. 10.1016/j.pharmthera.2024.108642 PMC12231147 38614254

[2] Zhang C, Xu C, Gao X, Yao Q. Platinum-based drugs for cancer therapy and anti-tumor strategies. Theranostics. 2022;12:2115–32. 35265202 10.7150/thno.69424 PMC8899578

[3] Davoudi M, Jadidi Y, Moayedi K, Farrokhi V, Afrisham R. Ameliorative impacts of polymeric and metallic nanoparticles on cisplatin-induced nephrotoxicity: a 2011-22 review. J Nanobiotechnol. 2022;20:504. 10.1186/s12951-022-01718-w PMC9714065 36457031

[4] Pickkers P, Darmon M, Hoste E, Joannidis M, Legrand M, Ostermann M, et al. Acute kidney injury in the critically ill: an updated review on pathophysiology and management. Intensive Care Med. 2021;47:835–50. 34213593 10.1007/s00134-021-06454-7 PMC8249842

[5] Tang C, Livingston MJ, Safirstein R, Dong Z. Cisplatin nephrotoxicity: new insights and therapeutic implications. Nat Rev Nephrol. 2023;19:53–72. 36229672 10.1038/s41581-022-00631-7

[6] Hamroun A, Lenain R, Bigna JJ, Speyer E, Bui L, Chamley P, et al. Prevention of cisplatin-induced acute kidney injury: a systematic review and meta-analysis. Drugs. 2019;79:1567–82. 31429065 10.1007/s40265-019-01182-1

[7] Sears SM, Siskind LJ. Potential therapeutic targets for cisplatin-induced kidney injury: lessons from other models of AKI and fibrosis. J Am Soc Nephrol. 2021;32:1559–67. 34049962 10.1681/ASN.2020101455 PMC8425641

[8] Pirozzi CJ, Yan H. The implications of IDH mutations for cancer development and therapy. Nat Rev Clin Oncol. 2021;18:645–61. 34131315 10.1038/s41571-021-00521-0

[9] Molenaar RJ, Maciejewski JP, Wilmink JW, van Noorden CJF. Wild-type and mutated IDH1/2 enzymes and therapy responses. Oncogene. 2018;37:1949–60. 29367755 10.1038/s41388-017-0077-z PMC5895605

[10] Ying M, You D, Zhu X, Cai L, Zeng S, Hu X. Lactate and glutamine support NADPH generation in cancer cells under glucose deprived conditions. Redox Biol. 2021;46:102065. 34293554 10.1016/j.redox.2021.102065 PMC8321918

[11] Chen L, Zhang Z, Hoshino A, Zheng HD, Morley M, Arany Z, et al. NADPH production by the oxidative pentose-phosphate pathway supports folate metabolism. Nat Metab. 2019;1:404–15. 31058257 PMC6489125

[12] Kadiyala P, Carney SV, Gauss JC, Garcia-Fabiani MB, Haase S, Alghamri MS, et al. Inhibition of 2-hydroxyglutarate elicits metabolic reprogramming and mutant IDH1 glioma immunity in mice. J Clin Invest. 2021;131:e139542. 33332283 10.1172/JCI139542 PMC7880418

[13] Tesileanu CMS, Vallentgoed WR, Sanson M, Taal W, Clement PM, Wick W, et al. Non-IDH1-R132H IDH1/2 mutations are associated with increased DNA methylation and improved survival in astrocytomas, compared to IDH1-R132H mutations. Acta Neuropathol. 2021;141:945–57. 33740099 10.1007/s00401-021-02291-6 PMC8113211

[14] Gondim DD, Gener MA, Curless KL, Cohen-Gadol AA, Hattab EM, Cheng L. Determining IDH-mutational status in gliomas using IDH1-R132H antibody and polymerase chain reaction. Appl Immunohistochem Mol Morphol. 2019;27:722–5. 30358614 10.1097/PAI.0000000000000702

[15] Ma L, Shi H, Li Y, Gao W, Guo J, Zhu J, et al. Hypertrophic preconditioning attenuates myocardial ischemia/reperfusion injury through the deacetylation of isocitrate dehydrogenase 2. Sci Bull (Beijing). 2021;66:2099–114. 36654268 10.1016/j.scib.2021.04.008

[16] Han YK, Kim JS, Lee GB, Lim JH, Park KM. Oxidative stress following acute kidney injury causes disruption of lung cell cilia and their release into the bronchoaveolar lavage fluid and lung injury, which are exacerbated by Idh2 deletion. Redox Biol. 2021;46:102077. 34315110 10.1016/j.redox.2021.102077 PMC8326422

[17] Han SJ, Choi HS, Kim JI, Park JW, Park KM. IDH2 deficiency increases the liver susceptibility to ischemia-reperfusion injury via increased mitochondrial oxidative injury. Redox Biol. 2018;14:142–53. 28938192 10.1016/j.redox.2017.09.003 PMC5608561

[18] Cleary JM, Rouaisnel B, Daina A, Raghavan S, Roller LA, Huffman BM, et al. Secondary IDH1 resistance mutations and oncogenic IDH2 mutations cause acquired resistance to ivosidenib in cholangiocarcinoma. NPJ Precis Oncol. 2022;6:61. 36056177 10.1038/s41698-022-00304-5 PMC9440204

[19] Reitman ZJ, Sinenko SA, Spana EP, Yan H. Genetic dissection of leukemia-associated IDH1 and IDH2 mutants and D-2-hydroxyglutarate in Drosophila. Blood. 2015;125:336–45. 25398939 10.1182/blood-2014-05-577940 PMC4287640

[20] Yu B, Jin L, Yao X, Zhang Y, Zhang G, Wang F, et al. TRPM2 protects against cisplatin-induced acute kidney injury and mitochondrial dysfunction via modulating autophagy. Theranostics. 2023;13:4356–75. 37649595 10.7150/thno.84655 PMC10465213

[21] Koppula P, Lei G, Zhang Y, Yan Y, Mao C, Kondiparthi L, et al. A targetable CoQ-FSP1 axis drives ferroptosis- and radiation-resistance in KEAP1 inactive lung cancers. Nat Commun. 2022;13:2206. 35459868 10.1038/s41467-022-29905-1 PMC9033817

[22] Secker PF, Schlichenmaier N, Beilmann M, Deschl U, Dietrich DR. Functional transepithelial transport measurements to detect nephrotoxicity in vitro using the RPTEC/TERT1 cell line. Arch Toxicol. 2019;93:1965–78. 31076804 10.1007/s00204-019-02469-8

[23] Xiang K, Wu H, Liu Y, Wang S, Li X, Yang B, et al. MOF-derived bimetallic nanozyme to catalyze ROS scavenging for protection of myocardial injury. Theranostics. 2023;13:2721–33. 37215581 10.7150/thno.83543 PMC10196836

[24] Oh CJ, Ha CM, Choi YK, Park S, Choe MS, Jeoung NH, et al. Pyruvate dehydrogenase kinase 4 deficiency attenuates cisplatin-induced acute kidney injury. Kidney Int. 2017;91:880–95. 28040265 10.1016/j.kint.2016.10.011

[25] Mapuskar KA, Pulliam CF, Tomanek-Chalkley A, Rastogi P, Wen H, Dayal S, et al. The antioxidant and anti-inflammatory activities of avasopasem manganese in age-associated, cisplatin-induced renal injury. Redox Biol. 2024;70:103022. 38215546 10.1016/j.redox.2023.103022 PMC10821164

[26] Cheng J, Zhu Y, Xing X, Xiao J, Chen H, Zhang H, et al. Manganese-deposited iron oxide promotes tumor-responsive ferroptosis that synergizes the apoptosis of cisplatin. Theranostics. 2021;11:5418–29. 33859755 10.7150/thno.53346 PMC8039957

[27] Lee J, You JH, Shin D, Roh JL. Inhibition of glutaredoxin 5 predisposes cisplatin-resistant head and neck cancer cells to ferroptosis. Theranostics. 2020;10:7775–86. 32685019 10.7150/thno.46903 PMC7359084

[28] Kim Y, Ju H, Yoo SY, Jeong J, Heo J, Lee S, et al. Glutathione dynamics is a potential predictive and therapeutic trait for neoadjuvant chemotherapy response in bladder cancer. Cell Rep Med. 2023;4:101224. 37797616 10.1016/j.xcrm.2023.101224 PMC10591055

[29] De Luca A, Parker LJ, Ang WH, Rodolfo C, Gabbarini V, Hancock NC, et al. A structure-based mechanism of cisplatin resistance mediated by glutathione transferase P1-1. Proc Natl Acad Sci USA. 2019;116:13943–51. 31221747 10.1073/pnas.1903297116 PMC6628828

[30] Cao X, Nie X, Xiong S, Cao L, Wu Z, Moore PK, et al. Renal protective effect of polysulfide in cisplatin-induced nephrotoxicity. Redox Biol. 2018;15:513–21. 29413963 10.1016/j.redox.2018.01.012 PMC5881418

[31] Newton K, Strasser A, Kayagaki N, Dixit VM. Cell death. Cell. 2024;187:235–56. 38242081 10.1016/j.cell.2023.11.044

[32] Gao M, Yi J, Zhu J, Minikes AM, Monian P, Thompson CB, et al. Role of mitochondria in ferroptosis. Mol Cell. 2019;73:354–63.e353. 30581146 10.1016/j.molcel.2018.10.042 PMC6338496

[33] Deng Z, Wang Y, Liu J, Zhang H, Zhou L, Zhao H, et al. WBP2 restrains the lysosomal degradation of GPX4 to inhibit ferroptosis in cisplatin-induced acute kidney injury. Redox Biol. 2023;65:102826. 37516014 10.1016/j.redox.2023.102826 PMC10410181

[34] Jiang X, Stockwell BR, Conrad M. Ferroptosis: mechanisms, biology and role in disease. Nat Rev Mol Cell Biol. 2021;22:266–82. 33495651 10.1038/s41580-020-00324-8 PMC8142022

[35] Shen K, Wang X, Wang Y, Jia Y, Zhang Y, Wang K, et al. miR-125b-5p in adipose derived stem cells exosome alleviates pulmonary microvascular endothelial cells ferroptosis via Keap1/Nrf2/GPX4 in sepsis lung injury. Redox Biol. 2023;62:102655. 36913799 10.1016/j.redox.2023.102655 PMC10023991

[36] Luo X, Wang Y, Zhu X, Chen Y, Xu B, Bai X, et al. MCL attenuates atherosclerosis by suppressing macrophage ferroptosis via targeting KEAP1/NRF2 interaction. Redox Biol. 2024;69:102987. 38100883 10.1016/j.redox.2023.102987 PMC10761782

[37] Yamamoto M, Kensler TW, Motohashi H. The KEAP1-NRF2 system: a thiol-based sensor-effector apparatus for maintaining redox homeostasis. Physiol Rev. 2018;98:1169–203. 29717933 10.1152/physrev.00023.2017 PMC9762786

[38] Adinolfi S, Patinen T, Jawahar Deen A, Pitkänen S, Härkönen J, Kansanen E, et al. The KEAP1-NRF2 pathway: targets for therapy and role in cancer. Redox Biol. 2023;63:102726. 37146513 10.1016/j.redox.2023.102726 PMC10189287

[39] Sun YY, Zhu HJ, Zhao RY, Zhou SY, Wang MQ, Yang Y, et al. Remote ischemic conditioning attenuates oxidative stress and inflammation via the Nrf2/HO-1 pathway in MCAO mice. Redox Biol. 2023;66:102852. 37598463 10.1016/j.redox.2023.102852 PMC10462885

[40] Liu Y, Wang S, Jin G, Gao K, Wang S, Zhang X, et al. Network pharmacology-based study on the mechanism of ShenKang injection in diabetic kidney disease through Keap1/Nrf2/Ho-1 signaling pathway. Phytomedicine. 2023;118:154915. 37392674 10.1016/j.phymed.2023.154915

[41] Li J, Lu K, Sun F, Tan S, Zhang X, Sheng W, et al. Panaxydol attenuates ferroptosis against LPS-induced acute lung injury in mice by Keap1-Nrf2/HO-1 pathway. J Transl Med. 2021;19:96. 33653364 10.1186/s12967-021-02745-1 PMC7927246

[42] Ahola S, Langer T. Ferroptosis in mitochondrial cardiomyopathy. Trends Cell Biol. 2024;34:150–60. 37419738 10.1016/j.tcb.2023.06.002

[43] Su L, Zhang J, Gomez H, Kellum JA, Peng Z. Mitochondria ROS and mitophagy in acute kidney injury. Autophagy. 2023;19:401–14. 35678504 10.1080/15548627.2022.2084862 PMC9851232

[44] Chakrabarty RP, Chandel NS. Mitochondria as signaling organelles control mammalian stem cell fate. Cell Stem Cell. 2021;28:394–408. 33667360 10.1016/j.stem.2021.02.011 PMC7944920

[45] Qiu S, Zhong X, Meng X, Li S, Qian X, Lu H, et al. Mitochondria-localized cGAS suppresses ferroptosis to promote cancer progression. Cell Res. 2023;33:299–311. 36864172 10.1038/s41422-023-00788-1 PMC10066369

[46] Zhong S, Chen W, Wang B, Gao C, Liu X, Song Y, et al. Energy stress modulation of AMPK/FoxO3 signaling inhibits mitochondria-associated ferroptosis. Redox Biol. 2023;63:102760. 37267686 10.1016/j.redox.2023.102760 PMC10244700

[47] Chuntova P, Yamamichi A, Chen T, Narayanaswamy R, Ronseaux S, Hudson C, et al. Inhibition of D-2HG leads to upregulation of a proinflammatory gene signature in a novel HLA-A2/HLA-DR1 transgenic mouse model of IDH1R132H-expressing glioma. J Immunother Cancer. 2022;10:e004644. 35606087 10.1136/jitc-2022-004644 PMC9174833

[48] Waitkus MS, Yan H. Targeting isocitrate dehydrogenase mutations in cancer: emerging evidence and diverging strategies. Clin Cancer Res. 2021;27:383–8. 32883741 10.1158/1078-0432.CCR-20-1827

[49] Chung C, Sweha SR, Pratt D, Tamrazi B, Panwalkar P, Banda A, et al. Integrated metabolic and epigenomic reprograming by H3K27M mutations in diffuse intrinsic pontine gliomas. Cancer Cell. 2020;38:334–49.e339. 32795401 10.1016/j.ccell.2020.07.008 PMC7494613

[50] Yu M, Hon GC, Szulwach KE, Song CX, Jin P, Ren B, et al. Tet-assisted bisulfite sequencing of 5-hydroxymethylcytosine. Nat Protoc. 2012;7:2159–70. 23196972 10.1038/nprot.2012.137 PMC3641661

[51] Yang J, Chen X, A L, Gao H, Zhao M, Ge L, Li M, Yang C, Gong Y, Gu Z, Xu H. Alleviation of photoreceptor degeneration based on fullerenols in rd1 mice by reversing mitochondrial dysfunction via modulation of mitochondrial DNA transcription and leakage. Small. 2023;19:e2205998. 37407519 10.1002/smll.202205998

[52] Okoye CN, Koren SA, Wojtovich AP. Mitochondrial complex I ROS production and redox signaling in hypoxia. Redox Biol. 2023;67:102926. 37871533 10.1016/j.redox.2023.102926 PMC10598411

[53] Guerrero-Castillo S, Baertling F, Kownatzki D, Wessels HJ, Arnold S, Brandt U, et al. The assembly pathway of mitochondrial respiratory chain complex I. Cell Metab. 2017;25:128–39. 27720676 10.1016/j.cmet.2016.09.002

[54] Jaeschke H, Adelusi OB, Akakpo JY, Nguyen NT, Sanchez-Guerrero G, Umbaugh DS, et al. Recommendations for the use of the acetaminophen hepatotoxicity model for mechanistic studies and how to avoid common pitfalls. Acta Pharm Sin B. 2021;11:3740–55. 35024303 10.1016/j.apsb.2021.09.023 PMC8727921

[55] Ma H, Guo X, Cui S, Wu Y, Zhang Y, Shen X, et al. Dephosphorylation of AMP-activated protein kinase exacerbates ischemia/reperfusion-induced acute kidney injury via mitochondrial dysfunction. Kidney Int. 2022;101:315–30. 34774556 10.1016/j.kint.2021.10.028

[56] Che Z, Zhou Z, Li SQ, Gao L, Xiao J, Wong NK. ROS/RNS as molecular signatures of chronic liver diseases. Trends Mol Med. 2023;29:951–67. 37704494 10.1016/j.molmed.2023.08.001

[57] Tonnus W, Meyer C, Steinebach C, Belavgeni A, von Mässenhausen A, Gonzalez NZ, et al. Dysfunction of the key ferroptosis-surveilling systems hypersensitizes mice to tubular necrosis during acute kidney injury. Nat Commun. 2021;12:4402. 34285231 10.1038/s41467-021-24712-6 PMC8292346

[58] Linkermann A, Skouta R, Himmerkus N, Mulay SR, Dewitz C, De Zen F, et al. Synchronized renal tubular cell death involves ferroptosis. Proc Natl Acad Sci USA. 2014;111:16836–41. 25385600 10.1073/pnas.1415518111 PMC4250130

[59] Li W, Liang L, Liu S, Yi H, Zhou Y. FSP1: a key regulator of ferroptosis. Trends Mol Med. 2023;29:753–64. 37357101 10.1016/j.molmed.2023.05.013

[60] Lv Y, Liang C, Sun Q, Zhu J, Xu H, Li X, et al. Structural insights into FSP1 catalysis and ferroptosis inhibition. Nat Commun. 2023;14:5933. 37739943 10.1038/s41467-023-41626-7 PMC10516921

[61] Bersuker K, Hendricks JM, Li Z, Magtanong L, Ford B, Tang PH, et al. The CoQ oxidoreductase FSP1 acts parallel to GPX4 to inhibit ferroptosis. Nature. 2019;575:688–92. 31634900 10.1038/s41586-019-1705-2 PMC6883167

[62] Lin X, Zhang Q, Li Q, Deng J, Shen S, Tang M, et al. Upregulation of CoQ shifts ferroptosis dependence from GPX4 to FSP1 in acquired radioresistance. Drug Resist Updat. 2024;73:101032. 38198846 10.1016/j.drup.2023.101032

[63] Li Y, Yuan Y, Huang ZX, Chen H, Lan R, Wang Z, et al. GSDME-mediated pyroptosis promotes inflammation and fibrosis in obstructive nephropathy. Cell Death Differ. 2021;28:2333–50. 33664482 10.1038/s41418-021-00755-6 PMC8329275

[64] Sasaki M, Knobbe CB, Munger JC, Lind EF, Brenner D, Brüstle A, et al. IDH1(R132H) mutation increases murine haematopoietic progenitors and alters epigenetics. Nature. 2012;488:656–9. 22763442 10.1038/nature11323 PMC4005896

[65] Lai K, Wang J, Lin S, Chen Z, Lin G, Ye K, et al. Sensing of mitochondrial DNA by ZBP1 promotes RIPK3-mediated necroptosis and ferroptosis in response to diquat poisoning. Cell Death Differ. 2024;31:635–50. 38493248 10.1038/s41418-024-01279-5 PMC11094118

