# Supplementary material for: The IDH1-R132H mutation aggravates cisplatin-induced acute kidney injury by promoting ferroptosis through disrupting NDUFA1 and FSP1 interaction
Source: Cell Death Differ. 2024 Sep 22;32(2):242–55. doi: 10.1038/s41418-024-01381-8 (PMC11802792; doi:10.1038/s41418-024-01381-8)
Supplement: Supplementary file 1 — Supplementary Materials [file 41418_2024_1381_MOESM1_ESM.pdf]

Online supplementary file for

The IDH1-R132H mutation aggravates cisplatin-induced acute kidney injury by promoting ferroptosis through disrupting NDUFA1 and FSP1 interaction

Lai, et al.
✉Correspondence to:
tmak@uhnres.utoronto.ca; xuyanfang99@hotmail.com

This PDF file includes:

Supplementary methods

Supplementary Figure 1-9 and legends

Supplementary methods

Mice

Ksp-Cre mice were generously provided by Professor Hui-Yao Lan from The Chinese University of Hong Kong [1]. These mice exhibit Cre recombinase expression specifically in renal tubules [2]. IDH1-LSL mice were sourced from Professor Tak Wah Mak at the University of Toronto. These mice carry a Lox-Stop-Lox (LSL) cassette inserted into the first intron of the gene encoding the IDH1-R132H mutant, rendering the DNA segment unable to produce wild-type or mutant IDH1 protein. These mice are designated as $Idh1^{LSL/LSL}$, while those with LSL inserted on only one DNA strand are designated as $Idh1^{WT/LSL}$. Crossbreeding of $Idh1^{WT/LSL}$ mice with $Idh1^{LSL/LSL}$ mice using Ksp-Cre mice results in excision of the LSL cassette at the Cre enzyme action site. This leads to the expression of the point mutation on that DNA strand, generating heterozygous $Idh1^{WT/Mut}$ or homozygous $Idh1^{Mut/Mut}$ mice specifically expressing the IDH1-R132H mutant protein in renal tubules. Given that IDH1-R132H mutations detected in vivo are predominantly heterozygous, we selected $Idh1^{WT/WT}Ksp^{Cre}$ and $Idh1^{WT/Mut}Ksp^{Cre}$ mice, respectively. Genotyping of mice was conducted via PCR experiments (**Fig. S9**). All mice were housed and bred in a specific pathogen-free (SPF) environment, with a 12-hour light/dark cycle, and provided ad libitum access to water and food. All experiments complied with the regulations for the care and use of laboratory animals in China.

Cell Viability and Cell death Assessment

For cell death assessment, cells were exposed to a staining solution containing 1 μg/ml propidium iodide (PI) and 2 μg/ml Hoechst-33342 for 10 minutes at 37°C. Subsequently, the

cells were promptly visualized utilizing a Leica DMi8 inverted microscope. Positive signals corresponding to PI and Hoechst were enumerated utilizing ImageJ software. To assess cell proliferation , the cell counting Kit-8 (MeilunBio, MA0218) was employed in accordance with the manufacturer's protocol.

Colony formation assay

IDH1$^{WT/WT}$ and *IDH1*$^{WT/Mut}$ cells were seeded into 12-well plates and incubated for 10 days. Subsequently, the cells were stained with crystal violet (C0121,Beyotime) for 30 min and then washed with phosphate buffered saline (PBS). After staining, the visible colonies were counted.

Analysis of intracellular reactive oxygen species (ROS) and mitochondrial ROS

For intracellular ROS detection, the cells were subjected to incubation with dichloro-dihydro- fluorescein diacetate (DCFH-DA, S0033S, Beyotime) at a final concentration of 10 μM, in FBS-free DMEM, at 37°C for 30 minutes in the absence of light. For mitochondrial ROS, cells were stained with Mito-tracker (C1035, Beyotime) and MitoSoxTM (M36009, ThermoFisher). Subsequently, they underwent two PBS washes. Following the manufacturer's instructions, the fluorescence signal was quantified by the confocal ZEISS LSM800 microscope or CytoFLEX cytometer instrument from Beckman Coulter.

Measurement of lipid peroxidation

Cells were initially seeded at a density of 1 × 105 cells per well in 12-well plates and allowed to incubate overnight. Subsequently, after the stipulated treatment durations with the compounds, the cells were trypsinized and suspended in 500 μl of PBS containing 10 μM C11-BODIPY 581/591 (Invitrogen, USA). This cell suspension was then incubated for 30

minutes at 37°C in an environment with 5% CO2. To assess lipid peroxidation, both green (excitation/emission: 488/510 nm) and red signals (excitation/emission: 581/610 nm) were concurrently acquired using the CytoFLEX 243 cytometer. A minimum of 10,000 single cells were analyzed for each well.

Measurement of mitochondrial morphology and mitochondrial membrane potential (MMP)

To assess mitochondrial morphology and mitochondrial membrane potential (MMP), MitoTracker and JC-10 staining were employed. Following the manufacturer's instructions, the cells were incubated with 100 nM MitoTracker or a 10 μM JC-10 (CA1310, Solarbio) staining solution for a period of 30 minutes at 37°C. Subsequently, they underwent two washes with HBSS and were imaged using a confocal microscope. For the JC-10 staining, the fluorescence signals corresponding to JC-10 monomer and aggregate were detected at excitation/emission wavelengths of 490/525 nm and 530/590 nm, respectively. To assess Malonaldehyde (MDA) Content in kidney tissues, the MDA Assay Kit (M496, DOJINDO) was employed in accordance with the manufacturer's protocol.

Duolink proximity ligation assay

Proximity ligation assays were conducted employing the Duolink system (obtained from Sigma- Aldrich). Cells were initially seeded in glass-bottom culture dishes for cultivation, subsequently treated with cisplatin for the designated time intervals, and then subjected to fixation using 4% paraformaldehyde for a duration of 30 minutes. This was followed by permeabilization using 0.5% Triton X-100 in PBS for 15 minutes. Samples were subjected to

blocking using Duolink blocking buffer for 1 hour at 37°C, followed by an incubation step with two primary antibodies (each at a 1:200 dilution) at 4°C overnight within a humidity chamber. Following the incubation period, samples underwent two consecutive 5-minute washes with wash buffer A, which consisted of 10 mM Tris-HCl, 150 mM NaCl, and 0.05% Tween 20. Anti-rabbit PLUS and anti-mouse MINUS PLA probes (obtained from Sigma-Aldrich, DUO92002 and DUO92004) were subsequently coupled to the primary antibodies for 1 hour at 37°C. Ligation and amplification steps were carried out using Duolink In Situ Detection Reagents Red (sourced from Sigma-Aldrich, DUO92008) within a humidity chamber at 37°C, with ligation taking place for 30 minutes and amplification for 100 minutes. Following amplification, the cells were subjected to a 10-minute wash in wash buffer B, comprised of 200 mM Tris-HCl (pH 7.5) and 100 mM NaCl. They were then counterstained with DAPI (C1002, Beyotime) at a 1:1000 dilution in wash Buffer B for 15 minutes, followed by a 1-minute rinse with 0.01% wash buffer B in water. Subsequently, images were captured using a confocal ZEISS LSM800 microscope.

Immunostaining and imaging

Cells were initially seeded in glass-bottom culture dishes and subsequently fixed with 4% paraformaldehyde for a duration of 30 minutes at room temperature. Following fixation, the samples were subjected to two rinses with PBS and then washed with PBS containing 0.25% Triton X-100 (0.25% PT) for 10 minutes. Subsequently, the samples were blocked using a solution of 10% goat serum in PBS supplemented with 0.05% Tween-20 (0.05% PBST) for 1 hour. This was followed by an overnight incubation with the primary antibody, which was

diluted at a ratio of 1:200 in a blocking buffer, all conducted at 4°C. The next day, the samples were subjected to a series of three washes with 0.05% PBST and subsequently incubated with the secondary antibody, which was diluted 1:200 in a blocking buffer, for a period of 45 minutes at room temperature. To stain the nuclei, DAPI was applied at room temperature for 15 minutes. Finally, images were captured utilizing a confocal ZEISS LSM800 microscope.

Histologic analysis of kidney sections

The kidneys were embedded in paraffin or optimal cutting temperature compound (OCT, 4538, Leica). Kidney sections were subjected to PAS staining to measure tubular injury. To assess cell death, the TUNEL (ApopTag Fluorescein In Situ Apoptosis Detection Kit) Assay Kit (Millipore) was employed in accordance with the manufacturer's protocol. For morphological quantifications, a minimum of 10 random visual fields under a microscope (magnification ×100) from kidney section were analyzed, consistent with our prior study [1, 3].

Ligase Independent Clone (LIC)

In LIC experiments, Exonuclease III is employed, possessing 35-55 exonuclease activity, capable of degrading blunt and 3' recessed ends but not 3' overhanging ends. LIC experiments utilize Exonuclease III's 3'→5' exonuclease activity to digest the ends of the vector and DNA fragments, exposing designed 15bp homologous arms, followed by E. coli cells utilizing their own repair mechanism to join complementary paired DNA fragments. The reaction system comprises: pBoB-N-Flag or pBoB-C-Flag vector (15-50ng), target fragment (50-100ng), 10X Exo III buffer (1μl), and ddH2O to a total volume of 10μl. After thorough mixing, the reaction

mixture is placed on ice for 10 minutes to reduce the temperature. Subsequently, 1μl of 20U Exonuclease III enzyme is added, and the reaction proceeds on ice for 60 minutes. Following this, 1μl of 0.5M EDTA is added to terminate the reaction, and the mixture is incubated in a water bath at 60°C for 5 minutes for enzyme inactivation. The tubes are then placed on ice for 5 minutes.

Bisμlfite sequencing PCR (BSP)

Genomic DNA of kidney tissue or PTEC from $Idh1^{WT/WT}Ksp^{Cre}$ and $Idh1^{WT/Mut}Ksp^{Cre}$ mice was extracted following the instructions of the Cell/Tissue Genomic DNA Extraction Kit (Cat# DP304). DNA was collected using a CB3 spin column and eluted with TE buffer. The DNA concentration was measured using a UV-visible spectrophotometer. Bisulfite conversion was performed according to the EZ DNA Methylation-Lightning Kit instructions. Briefly, 20 μl of DNA was mixed with 130 μl of Lightning Conversion Reagent, incubated in a PCR machine, and processed through multiple buffer treatments. The converted DNA was eluted with M-Elution Buffer. BSP primers targeting the methylated region of the NDUFA1 promoter were designed online at http://www.urogene.org/methprimer/index.html. Three pairs of primers were designed, each with a 15 bp homologous arm sequence. The PCR reaction mixture included bisulfite-converted DNA template, forward and reverse primers, DreamTaq Green PCR Master Mix, and ddH2O. The PCR conditions were as follows: initial denaturation at 95°C for 5 minutes, 36 cycles of 95°C for 30 seconds, 60°C for 30 seconds, and 72°C for 45 seconds, followed by a final extension at 72°C for 5 minutes and cooling at 12°C for 5 minutes. Finally, DNA electrophoresis and fragment recovery were performed.

Simplified Reduced Representation Bisulfite Sequencing (RRBS) Library preparation and sequencing

About 1μg of genomic DNA mixed with unmethylated lambda DNA was digested by MspI enzyme for 16 hours at 37°C. After digestion, the libraries were constructed as the Illumina Pair-End protocol with some modifications. Briefly, purified digested DNA was subsequently treated with a mix of T4 DNA polymerase, Klenow Fragment and T4 polynucleotide kinase to repair, blunt and phosphorylate ends. After that, the blunt DNA were 3' adenylated using Klenow Fragment (3'-5' exo-) and following with ligation to adaptors synthesized with 5'-methylcytosine instead of cytosine using T4 DNA Ligase. After each step, the DNA was purified using MinElute PCR Purification Kit (Qiagen). ZYMO EZ DNA Methylation-Gold Kit™ was employed to convert unmethylated cytosine into uracil according to the instructions. Finally, PCR was carried out in a final reaction volume of 50μl consisting of 20μl adapter ligated DNA, 4μl 2.5mM dNTP, 5μl 10×buffer, 0.5μl JumpStart™ Taq DNA Polymerase, 2μl PCR primers and 18.5μl water and the following thermal cycling program was 94°C 1 min, 12cycles of 94 °C 10s, 62°C 30s, 72°C 30 then prolong with 5min at 72°C and products could be hold at 12°C. Before analysis with Illumina sequencing platform, the size selected library was analyzed by the Bioanalyzer analysis system (Agilent, Santa Clara, USA) and quantified by the real time PCR. Bisulfite sequencing PCR

RBBS data analysis

Adapter sequences were trimmed by using cutadapt. The parameter settings used was " -a AGATCGGAAGAGC -m 35 -n 2". The cleaned reads were mapped back to genome using

BSMAP software version 2.90 (Xi & Li, 2009). The parameter settings used was “-n 0 -v 0.08 -g 1”. Methylation ratios were extracted from BSMAP output (SAM) using a Python script (methratio.py) which is distributed with the BSMAP package. Only unique mapped reads were used to calculate methylation ratios. Only cytosines in a CpG context with sufficient sequencing depth (greater than or equal to 5x coverage) were retained for further analysis. Differentially methylated regions (DMRs) were detected using metilene (Jühling et al., 2016) in de-novo mode among CpG sites with at least 5x coverage. The parameter settings used was “--mincpgs 5 --minMethDiff 0.1 --mtc 1 -X 1 -Y 1 -v 0.7”. Then detected DMR were filtered according to the standard: (1) Q-value must be less than 0.05; (2) methylation level difference must be greater than 0.1; (3) CpG number contained in DMR must be greater than 5; (4) the length of the DMR must be greater than 50bp.

Real-time quantitative polymerase chain reaction

Real-time quantitative polymerase chain reaction (RT-qPCR) was conducted according to the previous protocol [3, 4]. Primary proximal renal tubular epithelial cells (PTCs) were extracted according to the previous method [5]. RNA from PTCs and tubular epithelial cell were extracted separately using Trizol (Invitrogen, USA) reagent by the manufacturer's instructions and then transcribed into cDNA using a reverse transcriptase kit (Vazyme, R333-01). RT-qPCR was conducted using SYBR qPCR Mix as a fluorescent dye on QuantStudio 5 (Thermo Fisher Scientific). The primers involved in the current study were obtained from Sangon Biotech (Shanghai, China) and were listed as follows, NDUFA1 (Forward primer-5’ATGTGGTTCGAGATTCTCCCT-3’ and Reverse primer-5’-

TGGTACTGAACACGAGCAACT-3').

Co-immunoprecipitation assay

The HEK293T cells were harvested at 36 hours post-transfection and subsequently lysed in a lysis buffer composed of 1% Triton X-100 in PBS, supplemented with a protease inhibitor cocktail and a phosphatase inhibitor (obtained from New Cell & Molecular Biotech, P002). The lysate was maintained on ice for 10 minutes and then subjected to sonication for 2 minutes (at 20% power, with a 1-second on/3-second off cycle). Following centrifugation at 12,000 rpm for 30 minutes, the resulting supernatants were collected and combined with Anti-DYKDDDDK-Tag monoclonal antibody agarose-conjugated beads (purchased from Abmart, M20018) or Anti-HA Tag monoclonal antibody agarose-conjugated beads (purchased from Abmart, M20031). These mixtures were rotated at 4°C for a duration of 4 hours. Subsequently, the beads underwent a thorough washing process with lysis buffer, and the immunoprecipitated materials were eluted using PBS containing 200 μg/ml of 3X Flag Peptide (sourced from Beyotime, P9801) or HA peptide (sourced from Beyotime, P9808). The eluates were then combined with an equal volume of 2× SDS buffer in preparation for western blot analysis.

Generation of knockout cell lines using the CRISPR-Cas9 technique

The knockout of Ndufa1 and Fsp1 in cell lines was achieved through the utilization of the CRISPR-Cas9 system. Specifically, guide RNAs (sgRNAs) were cloned into the lentiviral lentiGuide vector. The targeted sequences for gRNA were as follows: 5'-CTCCCCGGACTCTCCGTCAT-3' for human *Ndufa1* and 5'-TGCACGTGGTGATCGTGGGC-3' for mouse *Fsp1*. Subsequently, the medium containing

the lentivirus was collected 48 hours post-transfection and employed to infect HK-2 or tubular epithelial cell (TECs) [6], with the addition of 10 μg/ml polybrene to facilitate the process. Following infection, the cells underwent selection using puromycin for a duration of 3 days. Subsequently, single cells were sorted into 96-well plates and cultured for a period of 3–4 weeks. Each colony was meticulously verified through sequencing and western blot analysis to confirm the successful deletion of the target gene.

Western blot analysis and antibodies

For kidney tissues, approximately 50 mg were placed in 400-500 μL radioimmunoprecipitation assay (RIPA) lysis buffer containing a cocktail of protease inhibitors and phosphatase inhibitor before homogenization. Protein concentrations were determined by the Bicinchoninic acid assay and then sodium dodecyl sulphate (SDS) was added. For cultured cells in vitro, cells were harvested and immediately lysed with 1.2×SDS gel-loading buffer. Lysates were then subjected to electrophoresis and separated by SDS polyacrylamide gel electrophoresis (SDS- PAGE), then transfer to polyvinylidene fluoride membranes (EMD Millipore). Membranes were blocked for 1 h in 5% BSA and incubated overnight at 4°C with the appropriate primary antibodies as indicated. After TBST (Tris-buffered saline with 0.1% Tween-20) washing, membranes were incubated with horseradish peroxidase-labeled secondary antibodies for 1h. The enhanced chemiluminescence (ECL) method was used to visualize the blots by a ChemiDoc Imaging System (BIO-RAD).

The antibodies employed in this study were as follows: anti-HO-1 (proteintech, 10701-1-AP), anti-FSP-1 (proteintech, 20886-1-AP), anti-xCT/SLC7A11 (HUABIO, HA600098),

anti-β-actin (HUABIO, EM21002), anti-NDUFA1 (proteintech, 15561-1-AP). Additionally, anti-ACSL4 (ab155282), anti-Gpx4 (ab125066), anti-Keap1 (ab227828), anti-NRF2 (ab31163), Goat Anti-Mouse IgG H&L (Alexa Fluor® 488) (ab150113), and Anti-4HNE (ab48506) were sourced from Abcam. Further reagents included anti-Flag (Abmart, M20008M), anti-DYKDDDDK- Tag mAb agarose conjugated beads (Abmart, M20018), Duolink® In Situ PLA® Probe Anti-Rabbit PLUS (Sigma Aldrich, DUO92002), Duolink® In Situ PLA® Probe Anti-Mouse MINUS (Sigma Aldrich, DUO92004). Additionally, we utilized a protease inhibitor cocktail and phosphatase inhibitor (New Cell & Molecular Biotech, P002)

Chemical reagents

Chemical substances employed in our experiments encompassed Cisplatin (MedChemExpress, HY-17394), Liproxstatin-1 (MedChemExpress, HY-12726), and Proteinase K (Sigma-Aldrich, 70663). Additionally, we utilized JC-10 (Solarbio, J8050); BODIPY 581/591 C11 (Invitrogen, D3861), Mitotracker (C1035), DAPI (C1002), Hoechst-33342 (C1022), and DCFH-DA (S0033S), all of which were sourced from Beyotime.

Lentivirus preparation and infection

To produce recombinant lentivirus, 293T cells were subjected to cotransfection with pBOB expression constructs and lentivirus-packing plasmids (PMDL/REV/VSVG) via calcium phosphate precipitation. Following the transfection, the medium containing the virus was collected approximately 40–45 hours later and subsequently administered to cells in the presence of 10 μg/ml polybrene. The infectious medium was replaced after 12–14 hours, and the infected cells were employed for experiments approximately 36–48 hours following

infection.

Statistical analyses

Results were represented at least three independently performed experiments. Statistical analysis was performed with Prism software (GraphPad Software, Inc.). The data are expressed as mean ± SD. Group comparisons were conducted using an unpaired t-test, and for multiple comparisons, a one-way ANOVA was employed, followed by post hoc Bonferroni correction. Statistical significance was attributed to differences with p-values < 0.05.

REFERENCES

1. Li Y, Yuan Y, Huang ZX, Chen H, Lan R, Wang Z*, et al.* GSDME-mediated pyroptosis promotes inflammation and fibrosis in obstructive nephropathy. *Cell death and differentiation* 2021, **28**(8)**:** 2333-2350.

2. Sasaki M, Knobbe CB, Munger JC, Lind EF, Brenner D, Brüstle A*, et al.* IDH1(R132H) mutation increases murine haematopoietic progenitors and alters epigenetics. *Nature* 2012, **488**(7413)**:** 656-659.

3. Chen H, Li Y, Wu J, Li G, Tao X, Lai K*, et al.* RIPK3 collaborates with GSDMD to drive tissue injury in lethal polymicrobial sepsis. *Cell death and differentiation* 2020, **27**(9)**:** 2568-2585.

4. Wang Y, Li Y, Chen Z, Yuan Y, Su Q, Ye K*, et al.* GSDMD-dependent neutrophil extracellular traps promote macrophage-to-myofibroblast transition and renal fibrosis in obstructive nephropathy. *Cell death & disease* 2022, **13**(8)**:** 693.

5. Xu Y, Ma H, Shao J, Wu J, Zhou L, Zhang Z*, et al.* A Role for Tubular Necroptosis in Cisplatin-Induced AKI. *Journal of the American Society of Nephrology : JASN* 2015, **26**(11)**:** 2647-2658.

6. Lai K, Wang J, Lin S, Chen Z, Lin G, Ye K*, et al.* Sensing of mitochondrial DNA by ZBP1 promotes RIPK3-mediated necroptosis and ferroptosis in response to diquat poisoning. *Cell death and differentiation* 2024, **31**(5)**:** 635-650.

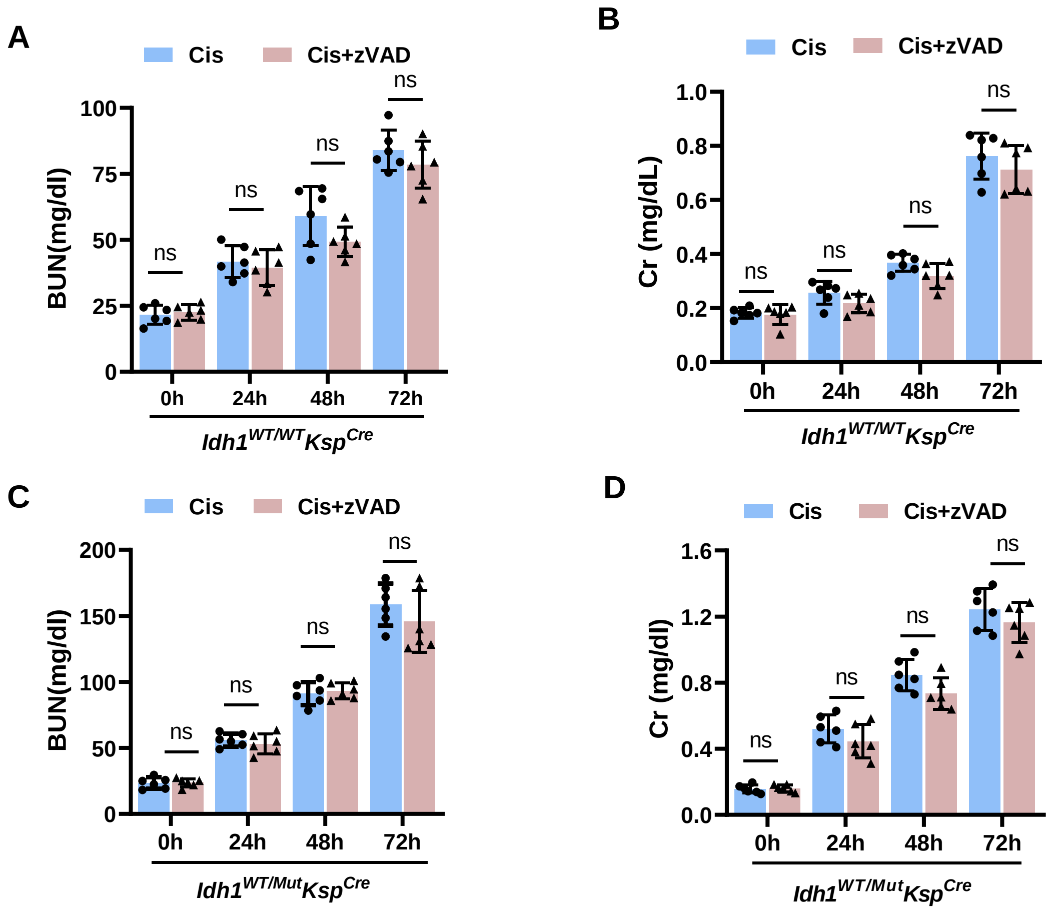

Fig. S1 The apoptosis inhibitor zVAD fails to ameliorate cisplatin-induced renal dysfunction in mice with the IDH1-R132H mutation.

(A-D). Blood urea nitrogen (BUN) (A,C) and serum creatinine (Scr) levels (B, D) were measured at 0, 24, 48, and 72 hours after cisplatin administration. Mice with *Idh1*$^{WT/Mut}$*Ksp*^{Cre} showed higher Scr and BUN levels compared to the *Idh1*$^{WT/WT}$*Ksp*^{Cre} group with better renal function. Both *Idh1*$^{WT/WT}$*Ksp*^{Cre} and *Idh1*$^{WT/Mut}$*Ksp*^{Cre} mice were pretreated with zVAD before intraperitoneal injection of 10 mg/kg cisplatin. The pretreatment with zVAD (5mg/kg) failed to improve renal function at any of the time points in both *Idh1*$^{WT/WT}$*Ksp*^{Cre} and *Idh1*$^{WT/Mut}$*Ksp*^{Cre} mice. ns = not significant.

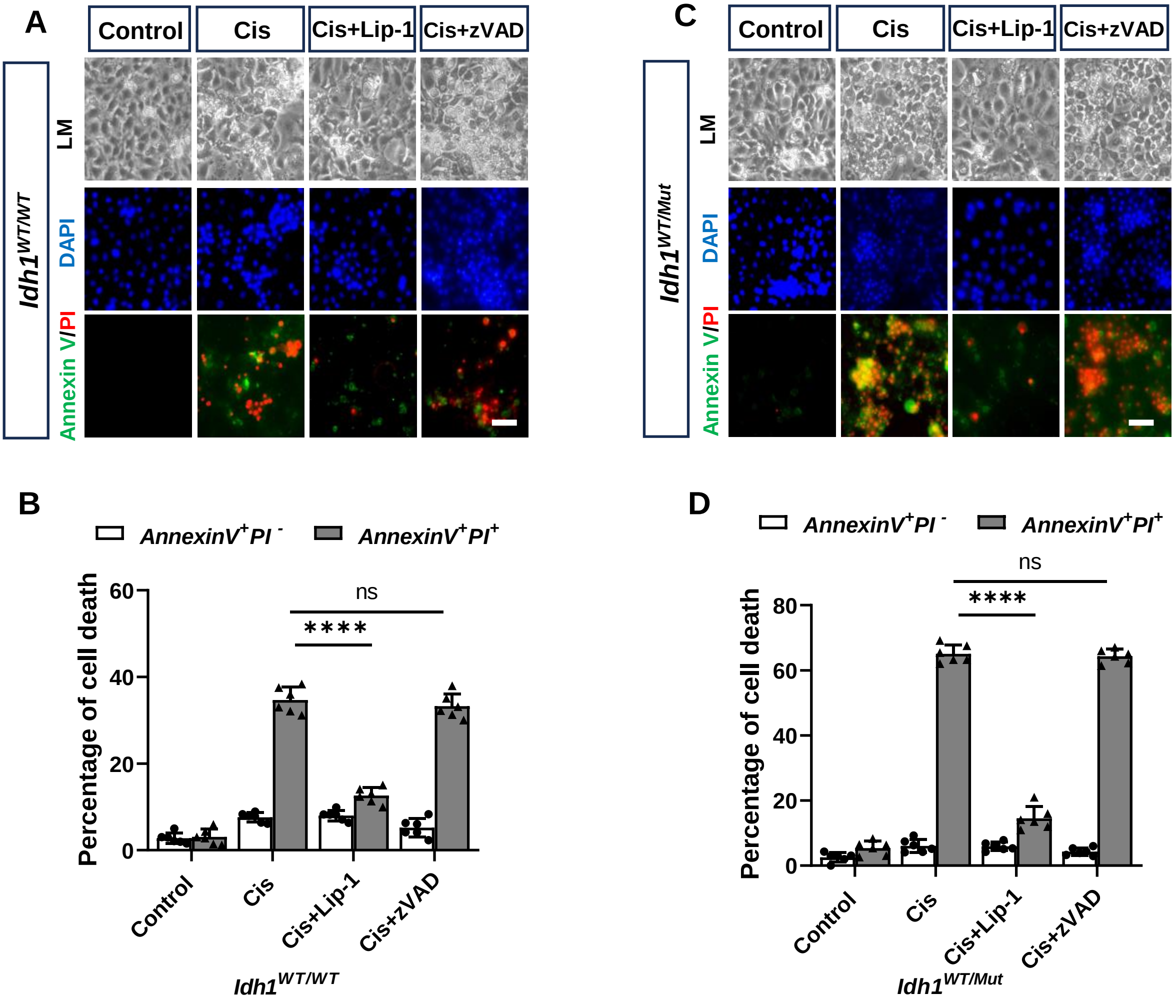

Fig. S2 The apoptosis inhibitor zVAD fails to prevent necrotic cell death compared to Lip-1.

A-D. Reprsentative staining images and quantification of *Annexin* V^{+} PI^{-} and *Annexin* $V^{+}PI^{+}$ cells in $Idh^{WT/WT}$ and $Idh^{WT/Mut}$ groups . *n*=6. ns = not significant; *$P < 0.05$; **$P < 0.01$; ***$P < 0.001$; ****$P < 0.0001$. Scale bar = 100 μm

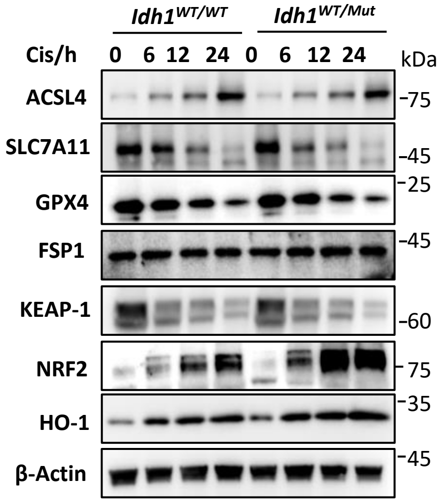

Fig. S3 Western blot analysis of ferroptotic-related proteins in in *vitro* cultured PTCs.

Western blot analysis of ferroptotic and antioxidant signaling molecules in *vitro* cultured *Idh1*$^{WT/WT}$ and *Idh1*$^{WT/Mut}$ PTCs isolated from mice of different genotypes after cisplatin treatment, n = 4.

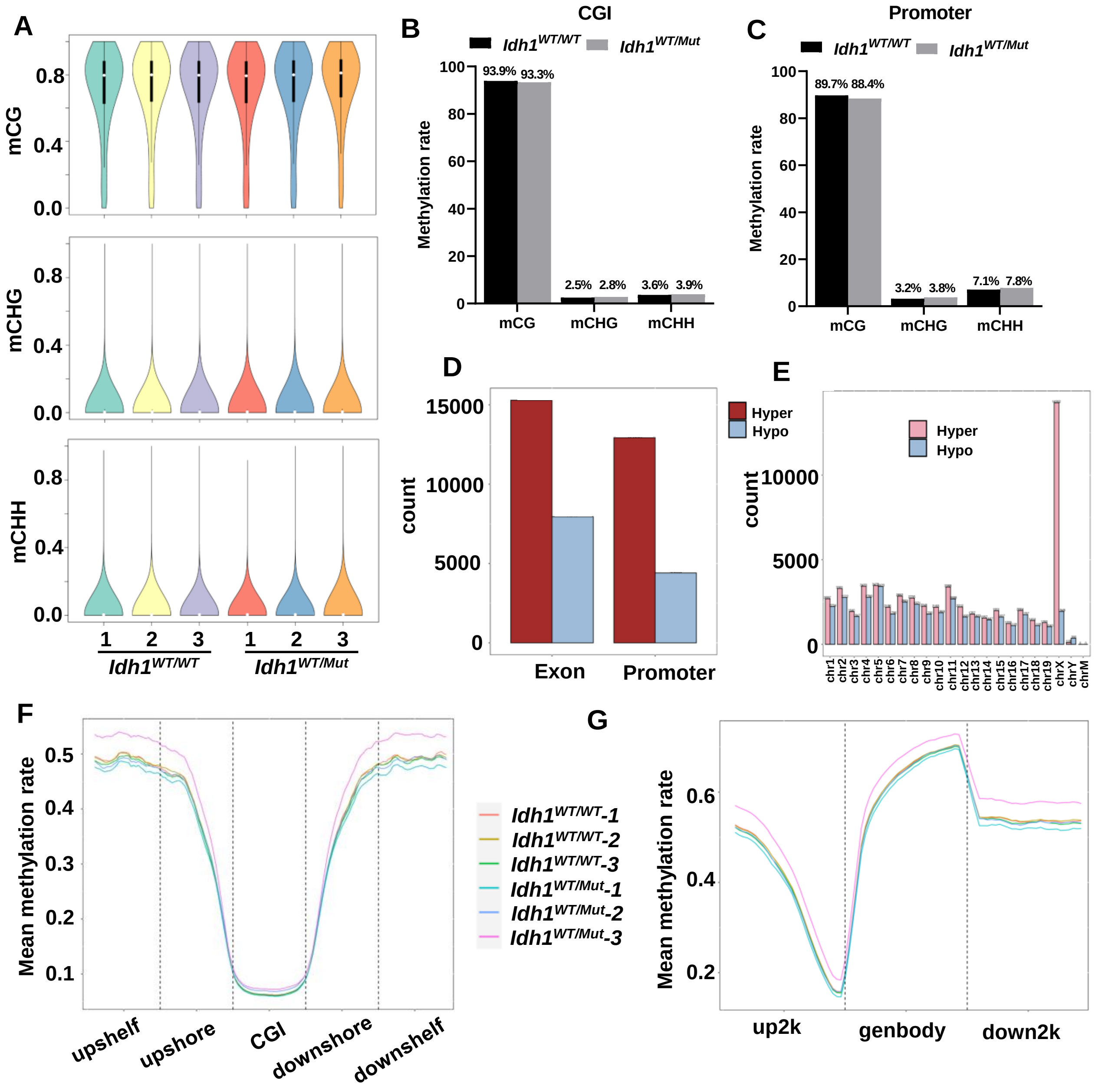

Fig. S4 Quality control of Reduced Representation Bisulfite Sequencing (RBBS).
(A). Distribution characteristics of methylation levels across six samples used for RBBS sequencing, showing consistent distribution of CG, CHG, and CHH methylation sites across different samples. (B, C). Methylation rates of CG, CHG, and CHH in CpG islands and DNA promoter regions in *Idh1*$^{WT/WT}$ and *Idh1*$^{WT/Mut}$ groups. mCG, mCHG, and mCHH represent the methylated forms of CG, CHG, and CHH, respectively. (D, E). Distribution of differentially methylated sites in gene elements and across chromosomes.F,G Methylation level distribution of genic elements. CGI: CpG island. downshelf: CGI upstream 2-4kb. downshore: CGI upstream 2kb. upshelf：CGI up stream 2-4kb. upshore：CGI upstream 2kb. up2k：2k upstream of the start site of gene transcription. genebody：Gene region. down2k：2K downstream of gene.

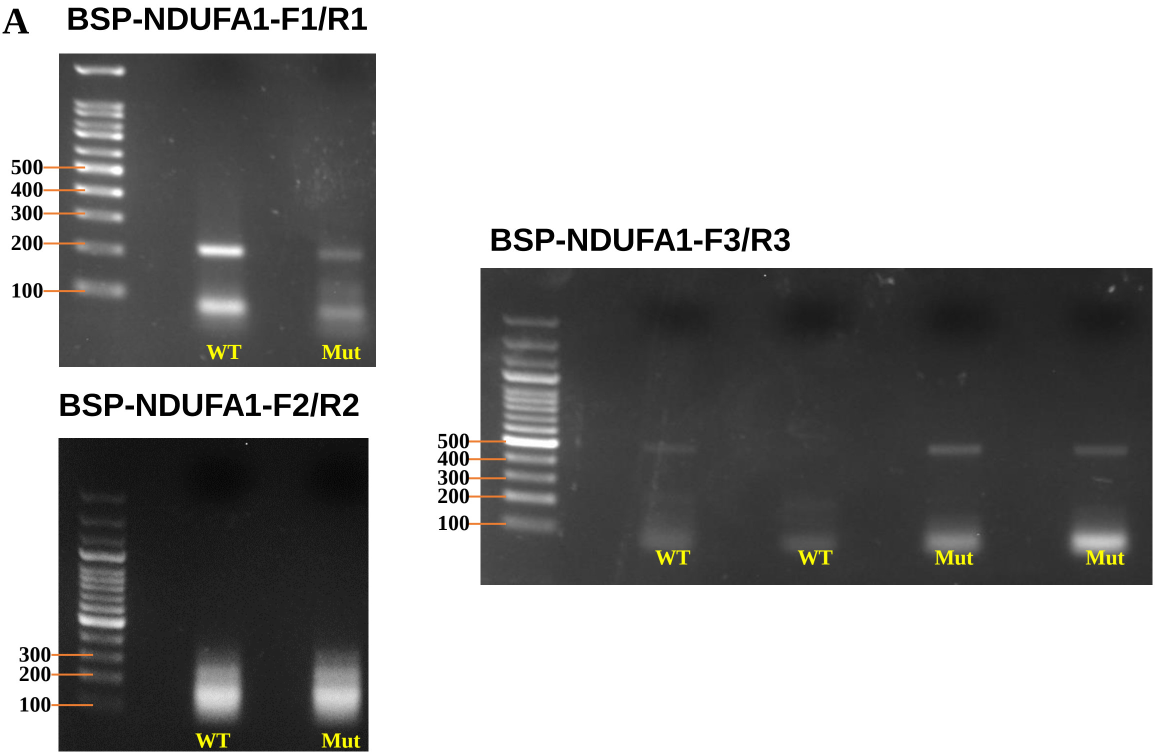

Fig. S5 The methylation level of the *Ndufa1* promoter in the PTCs of $\boldsymbol{Idh1^{WT/Mut}Ksp^{Cre}}$ mice is significantly higher than that in the PTCs of $\boldsymbol{Idh1^{WT/WT}Ksp^{Cre}}$ mice.

(A) Electrophoresis image of BSP amplification of *Ndufa1* promoter.

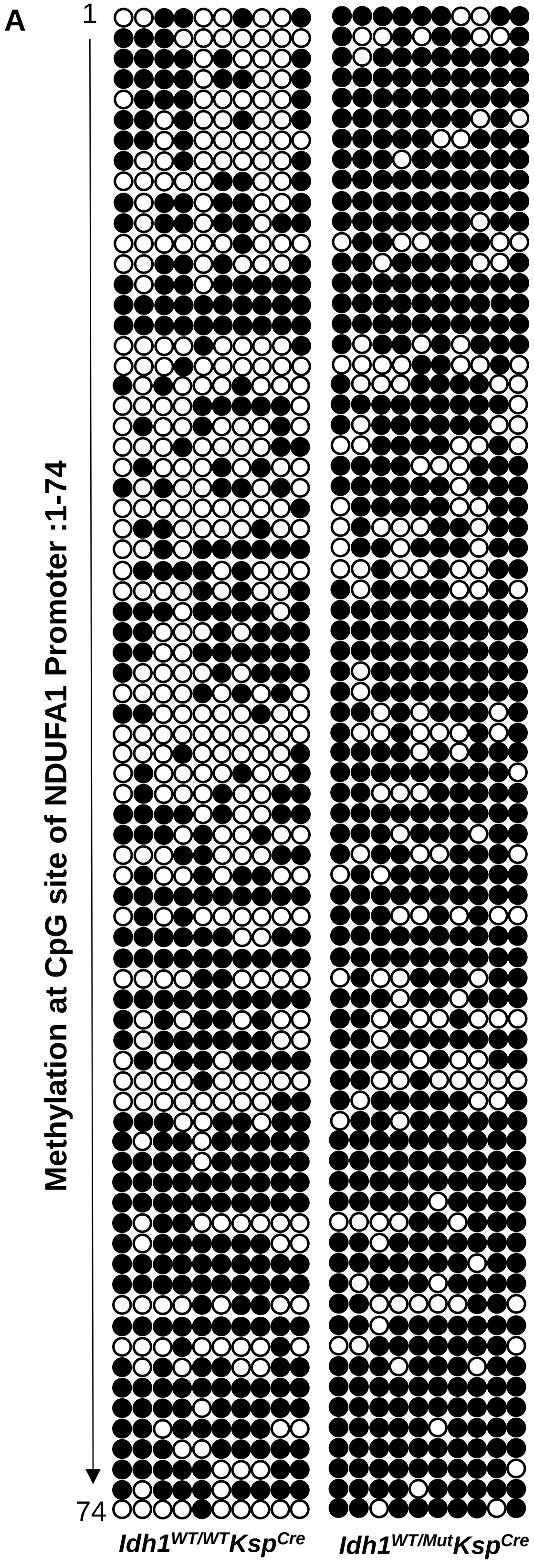

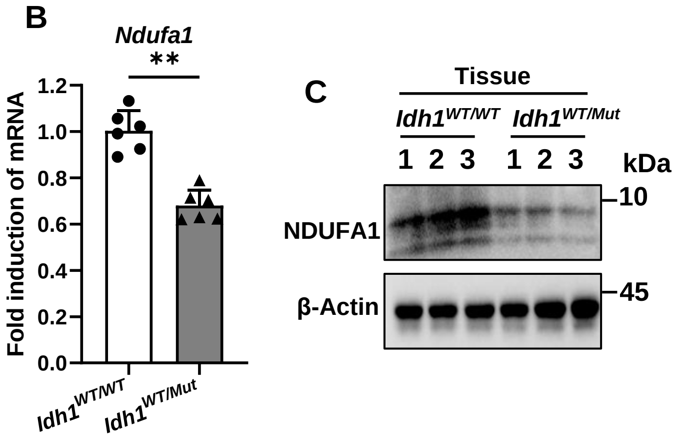

Fig. S6 The methylation level of the *Ndufa1* promoter in the kidneys of *Idh1$^{WT/Mut}$Ksp^{Cre}* mice is significantly higher than that in the kidneys of *Idh1$^{WT/WT}$Ksp^{Cre}* mice.

(A) Approximately 54% of CpG sites in this segment of *Ndufa1* were methylated in the kidney tissue of the *Idh1$^{WT/WT}$Ksp^{Cre}* mice, while in *Idh1$^{WT/Mut}$Ksp^{Cre}* group, approximately 80% of CpG sites were methylated.

(B, C) QT-PCR (B) and western blot (C) analyses in vivo indicated a decrease in the expression levels of NDUFA1.

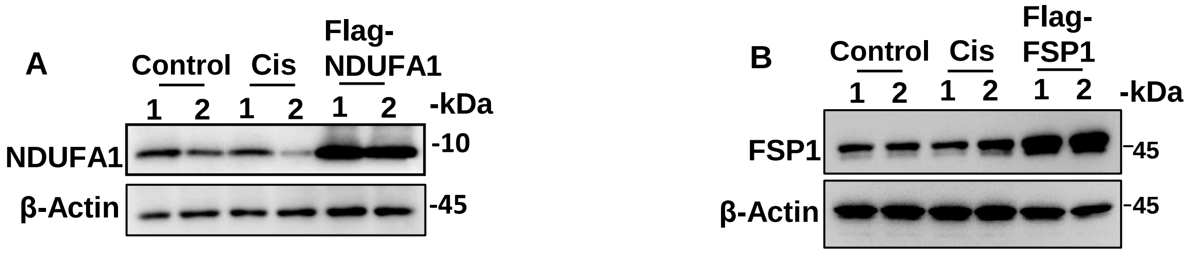

Fig. S7 Validation of NDUFA1 and FSP1 overexpression in PTCs using western blot

(A) Western blot analysis of PTCs overexpressing NDUFA1. (B) Western blot analysis of PTCs overexpressing FSP1.

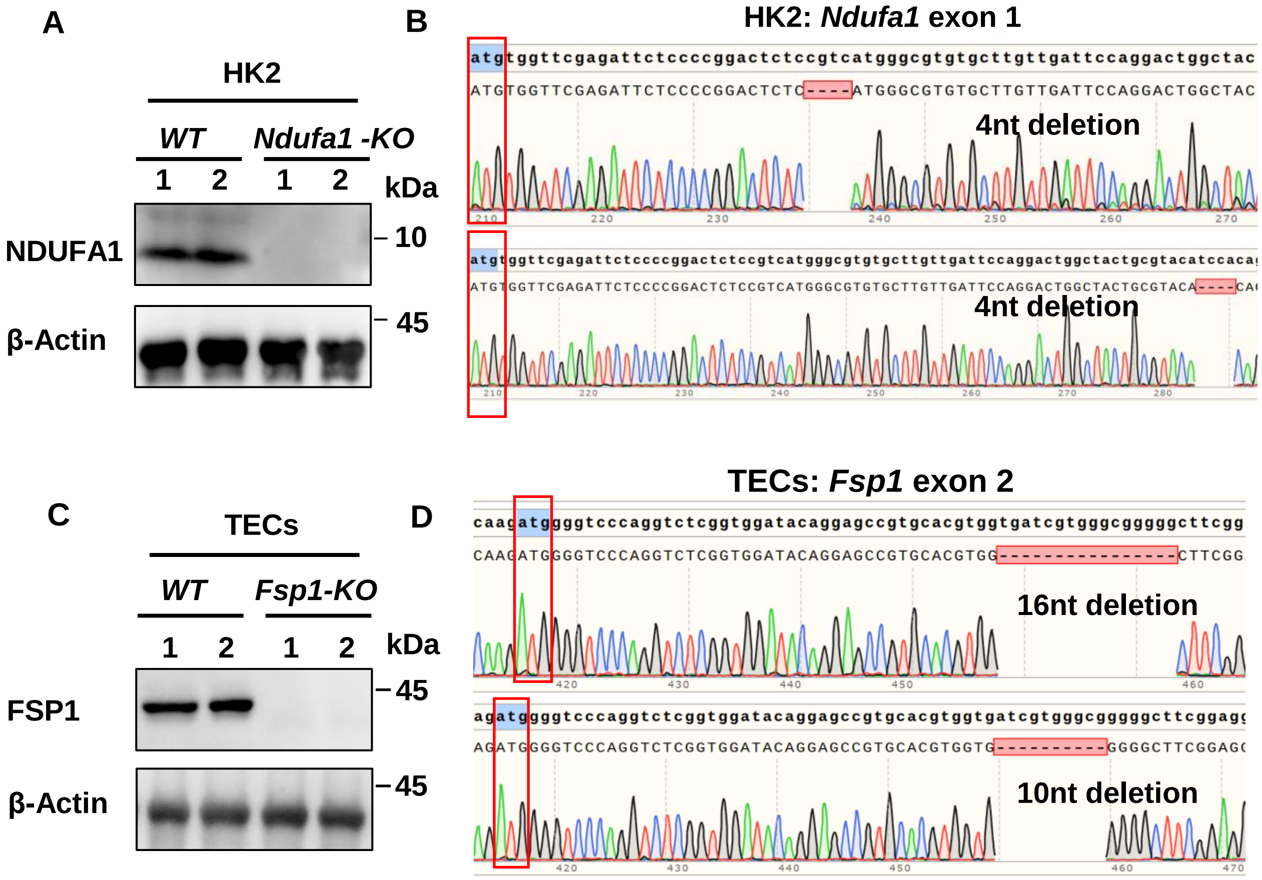

Fig. S8 Construction and Validation of Ndufa1 and Fsp1 Knockout Cell Lines
(A) The knockout efficiency of *Ndufa1 in* HK-2 cells was confirmed by western blot. (B) Genomic sequencing of the *Ndufa1* gene was performed in *Ndufa1-KO* HK-2, with the boxed region highlighting the ATG start codon. (C)The knockout efficiency of *Fsp1* in mouse tubular epithelial cells (TECs) was confirmed by western blot. (D) Genomic sequencing of the *Fsp1* gene was performed in *Fsp1-KO* TECs, with the boxed region highlighting the ATG start codon.

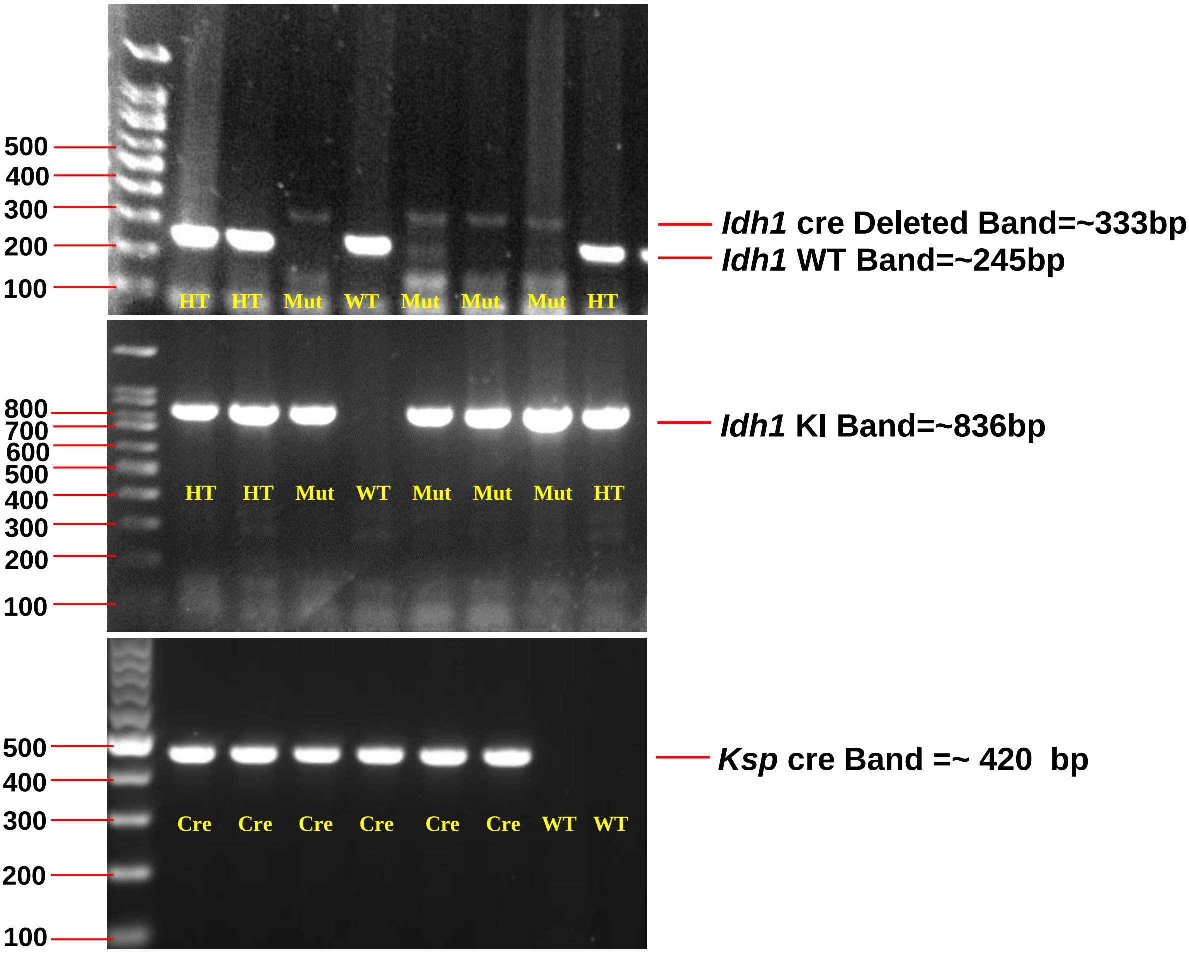

Fig. S9. Genotyping of kidney tubule-specific IDH1-R132H knock-in Mice.
The *Idh1* cre-deleted band (333 bp) indicates the deletion of the LSL locus in the *Idh1* gene mediated by cre recombinase. The *Idh1* KI band (836 bp) indicates the knock-in of the *Idh1* gene. The *Idh1* WT band (245 bp) indicates the wild-type (WT) genotype of the *Idh1* gene, with no insertions or deletions. The *Ksp* cre band (420 bp) indicates the expression of a specific gene in the kidneys of mice. If the PCR results show both the *Idh1* cre-deleted band (333 bp) and the *Idh1* KI band (836 bp), it suggests that both alleles of the mouse are LSL loci. If the results show both the *Idh1* WT band (245 bp) and the *Idh1* KI band (836 bp), it indicates that the mouse is heterozygous (HT), with one allele being the LSL locus and the other being the WT allele. If only the *Idh1* WT band (245 bp) is detected and the *Idh1* KI band is absent, it indicates that both alleles of the mouse are WT. After determining the genotype of the mice through PCR, the appropriate mice are selected for subsequent experiments.
