# Supplementary material for: The IDH1-R132H mutation aggravates cisplatin-induced acute kidney injury by promoting ferroptosis through disrupting NDUFA1 and FSP1 interaction
Source: Cell Death Differ. 2024 Sep 22;32(2):242–55. doi: 10.1038/s41418-024-01381-8 (PMC11802792; doi:10.1038/s41418-024-01381-8)

Online supplementary file for

The IDH1-R132H mutation aggravates cisplatin-induced acute kidney injury by promoting ferroptosis through disrupting NDUFA1 and FSP1 interaction

Lai, et al.

✉Correspondence to:

tmak@uhnres.utoronto.ca; xuyanfang99@hotmail.com

This PDF file includes:

Supplementary original western blots gel

Fig. 2F

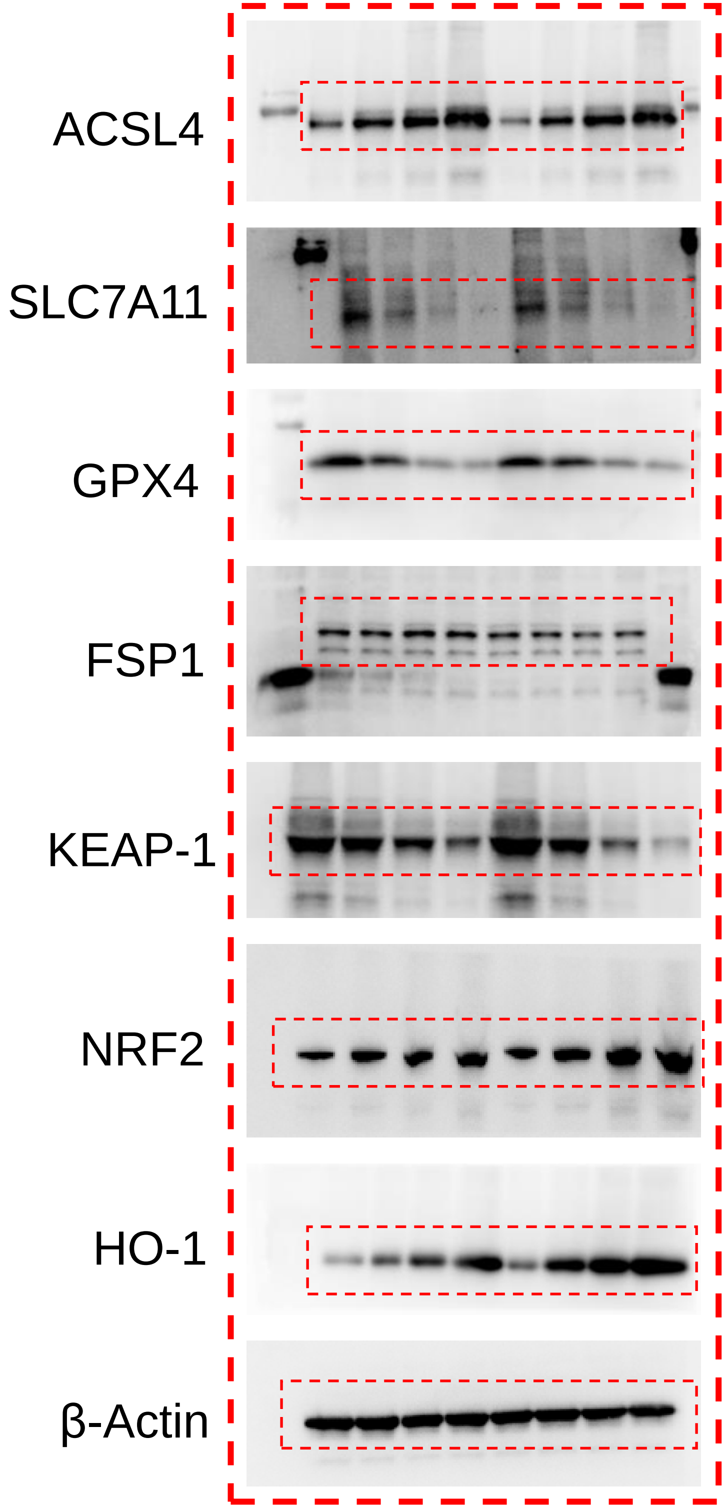

Fig. 3A

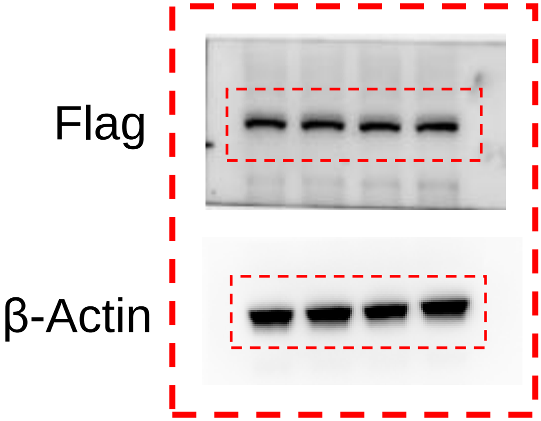

Fig. 4D

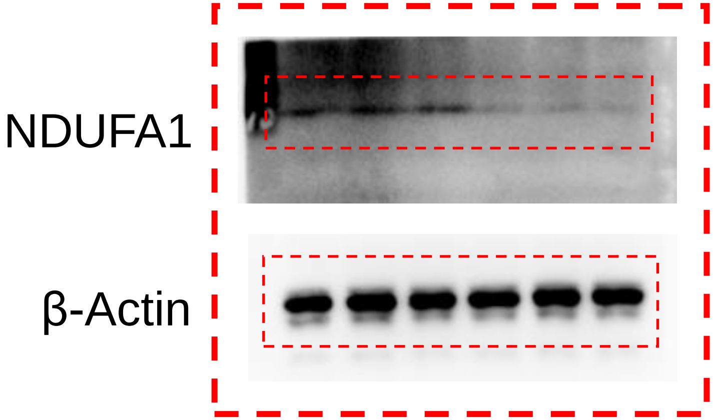

Fig. 6A

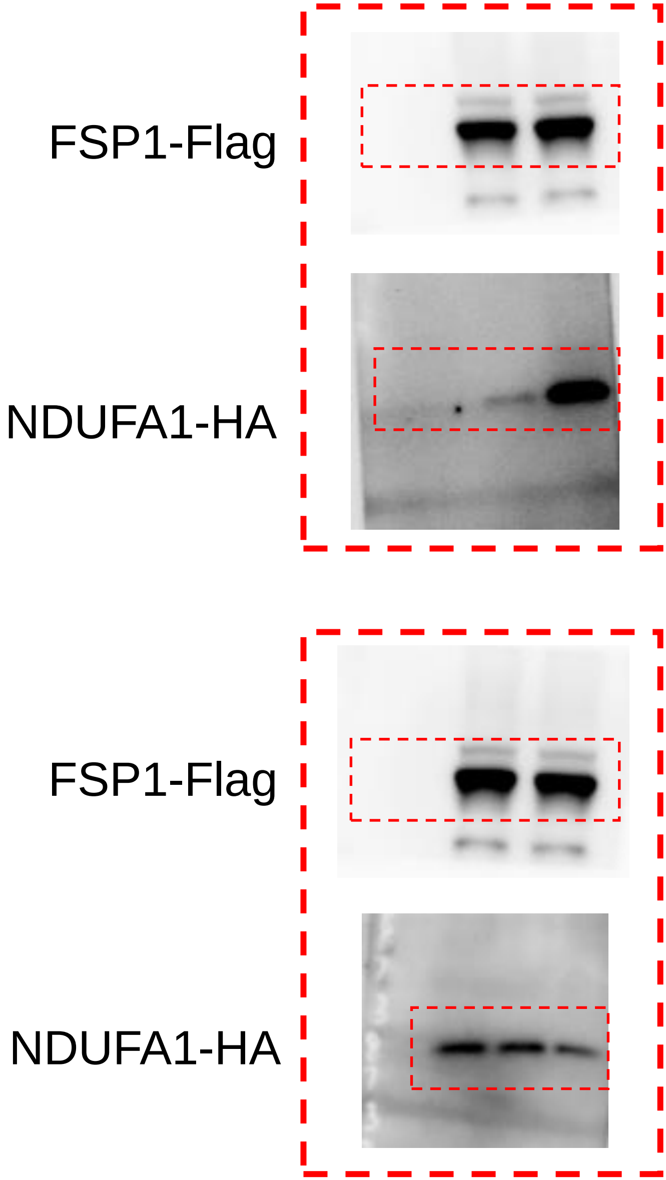

Fig. S3

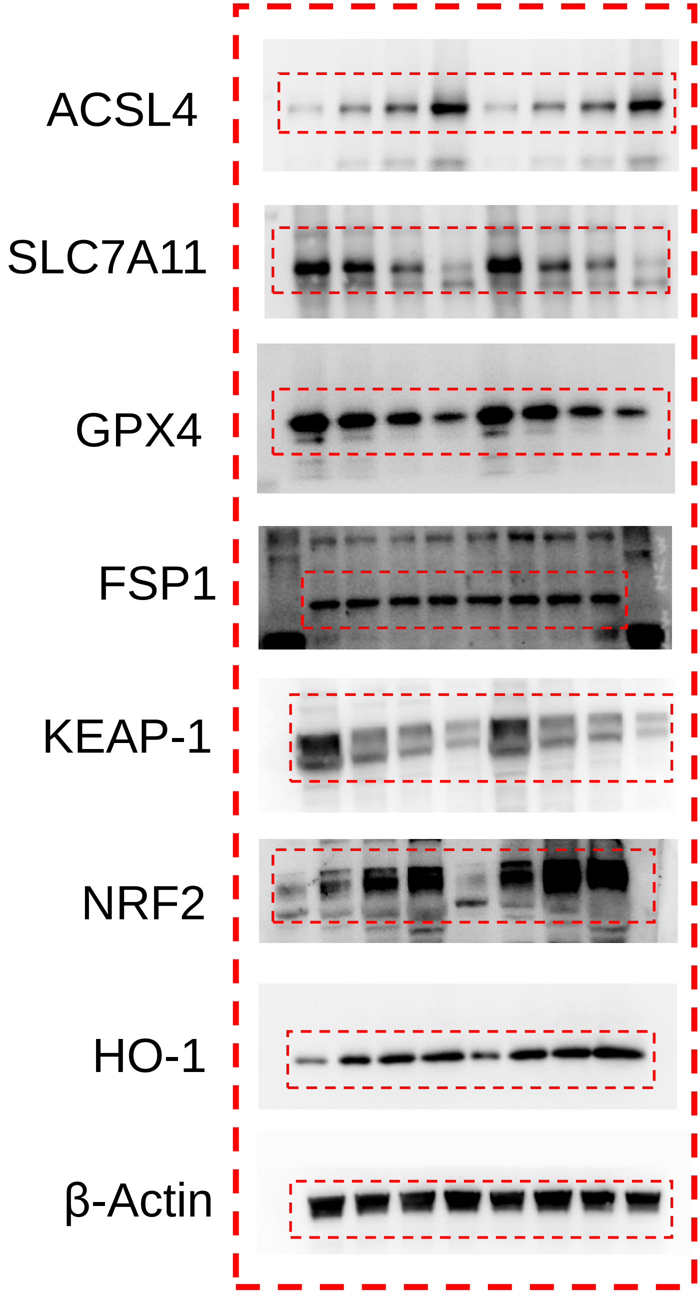

Fig. S6C

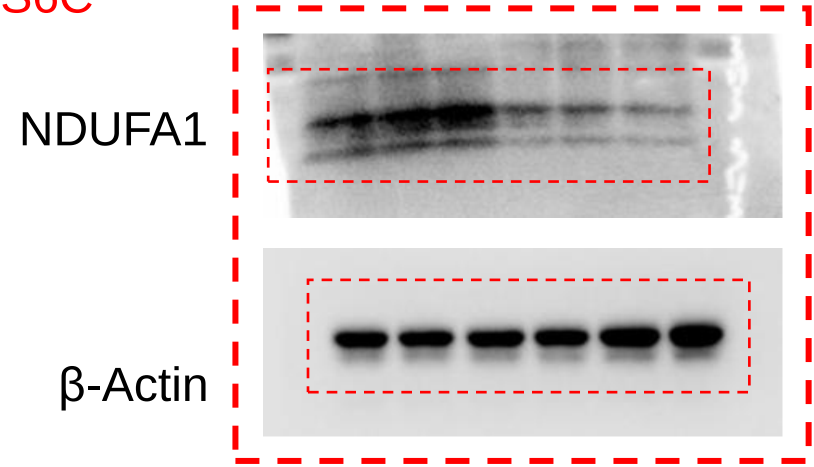

Fig. S7A

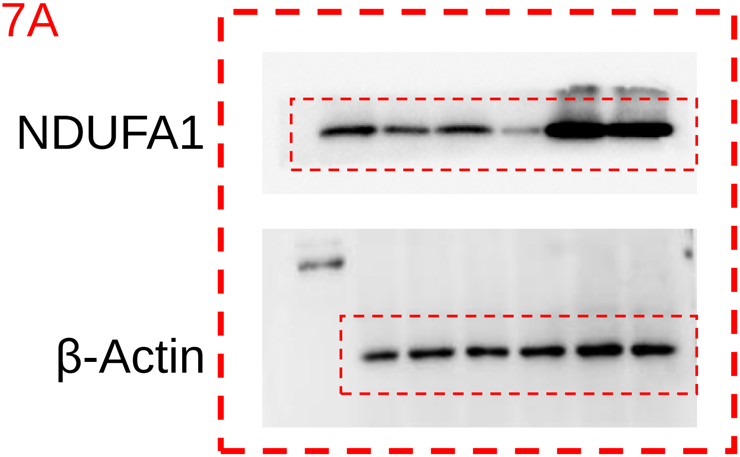

Fig. S7B

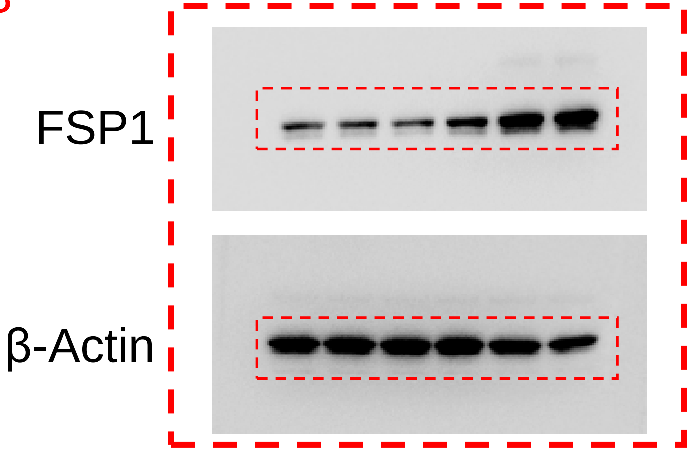

Fig. S8A

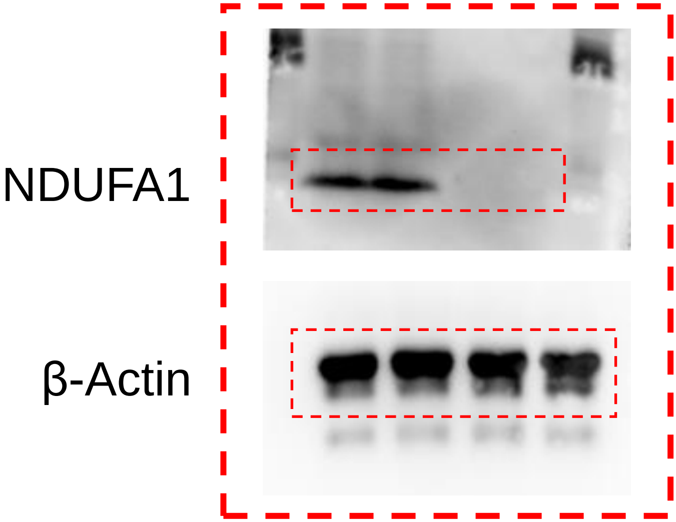

Fig. S8C

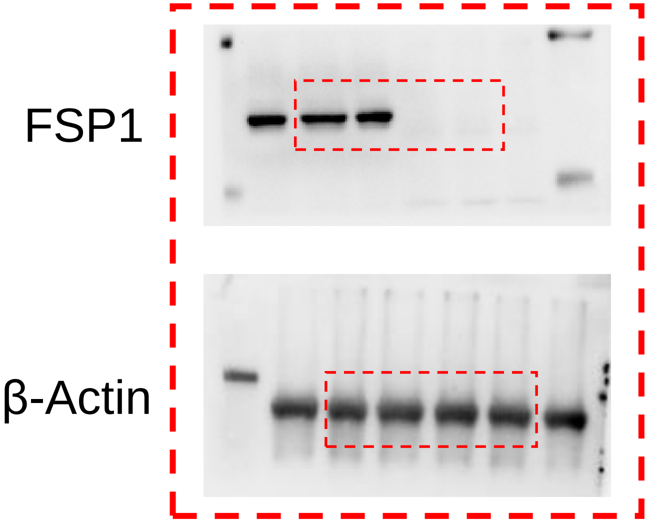

Supplement: Supplementary file 2 — Supplementary original western blots gel [file 41418_2024_1381_MOESM2_ESM.pdf]
